# Efficacy and Safety of a Mineral Oil-Based Head Lice Shampoo: A Randomized, Controlled, Investigator-Blinded, Comparative Study

**DOI:** 10.1371/journal.pone.0156853

**Published:** 2016-06-10

**Authors:** Luise Wolf, Frank Eertmans, Doerte Wolf, Bart Rossel, Els Adriaens

**Affiliations:** 1 Cardiosec Clinical Research GmbH, Dalbergsweg 21, 99084, Erfurt, Germany; 2 Oystershell Laboratories, Booiebos 24, 9031, Drongen, OVL, Belgium; 3 Adriaens Consulting, Bellemdorpweg 95, 9881, Bellem, OVL, Belgium; Azienda ospedaliero-universitaria di Perugia, ITALY

## Abstract

**Background:**

Due to increased resistance and safety concerns with insecticide-based pediculicides, there is growing demand for head lice treatments with a physical mode of action. Certain mineral oils kill lice by blocking spiracles or by disrupting the epicuticular wax layer. The present study was performed to evaluate efficacy and safety of a mineral oil-based shampoo.

**Methods:**

This randomized, controlled, investigator-blinded, monocentric study (EudraCT registration no. 2014-002918-23) was performed from October 2014—June 2015 in Germany. A mineral oil shampoo (Mosquito^®^ Med Läuse Shampoo 10 in Germany, Paranix or Silcap shampoo elsewhere), registered as medical device, was compared to a conventional, locally reimbursed, pyrethroid-based pediculicide (Goldgeist^®^ Forte solution). In total, 107 patients (>1 year) with confirmed head lice infestation were included (test arm: n = 53; control arm: n = 54). All subjects received two applications of either test or control product at day 0 and day 7, according to the instructions for use. Efficacy and safety was evaluated directly, 1h and 24h after first application, before and after second treatment, and at day 10. The main objective was demonstrating a cure rate for the test product, being superior to 70% at day 10.

**Results:**

Cure rates at day 10 (corrected for re-infestation) for the test product (96.1%) and control (94%) significantly exceeded the pre-defined target (70%) (p < 0.001, 2-sided, 1-sample, chi-square test) with confirmed non-inferiority for the test product. Over all visits, cure rates were consistently higher for the test product, whereas more initially-cured subjects remained lice-free until end of study (78%; control: 60%). Both products were safe and well tolerated, offering good esthetical effects.

**Conclusion:**

This study showed that substance-based medical devices (including the tested mineral oil shampoo) can be safe and effective alternatives for insecticide-based pediculicides, with less risk for development of resistance because of the physical mode of action.

**Trial Registration:**

German Clinical Trials Register (DRKS) DRKS00009753 and EudraCT database 2014-002918-23

## Introduction

Head lice (*Pediculus humanus capitis*) are small (2–4 mm), hematophagous ectoparasites which live exclusively on the human scalp. They are spread by direct contact with an infested individual, especially by head-to-head contact. Head lice can infest all types of hair, and it is a common nuisance, particularly in school-aged children. Population-based studies in European countries show highly diverging prevalence values, ranging from 0.45% to 22.4% [1, 2].

Treatment of head lice infestations remains a stubborn problem in families. Previously, only insecticide-based products (e.g. pyrethroids) were available as treatment but they have been overtaken by physically acting, substance-based medical devices. In other regions of the world such as the United States and East Asia, conventional insecticides still lead the market. However, increasing resistance to these neurotoxic agents and raising safety concerns [3–7] play in favour of products with a physical mode of action.

For over a century, petroleum-derived mineral oils (so-called suffocating oils), have been used as topical human and veterinary insecticides and as plant disease control agents, respectively. Their physical mode of action is widely accepted and is based on covering and suffocation of ectoparasites such as mites and lice [8, 9].

During the last decades, purification processes have been further optimized, yielding highly refined and pure mineral oils. These oils have been approved by different competent authorities for horticultural, cosmetic as well as pharmaceutical use.

Covering head lice with mineral oils will result in respiratory spiracle blockage, which in turn will disrupt water and gas exchange. It is also suggested that mineral oils interfere with the insects’ epicuticular wax layer. These physical interactions finally result in mortality of adult lice and their nits [8–11].

The present study aimed to robustly evaluate the efficacy and safety of a mineral oil head lice shampoo (known in Germany as Mosquito^®^ Läuse Shampoo 10 and in other countries as Paranix or Silcap shampoo) and compare it to a traditional and locally reimbursed, pyrethroid-based pediculicide: Goldgeist^®^ Forte solution.

## Methods

### Study Set-up

This randomized, controlled, investigator-blinded, comparative study was approved by the ethics committee of the Medical chamber of the German federal state of Thuringia and by the German national health authority (BfArM). The Ethics committee approved the study on 8^th^ August 2014. The study was conducted in accordance with the principles of the Declaration of Helsinki 2013, Good Clinical Practice, and of the European Union Directive 2001/20/EC as well as the requirements of German drug law and data protection laws. The trial was registered to the EudraCT database (EudraCT No. 2014-002918-23). After recruitment was complete, the trial was additionally registered in the “German Clinical Trials Register (GermanCTR)” to make it publicly available (registration number: DRKS00009753). The authors confirm that all ongoing and related trials for this intervention have been registered.

The entire study took place at a clinical trial facility, specialized in conducting clinical studies with children. Recruitment was performed by trained personnel and continued from 13^th^ October 2014 (first patient first visit) to 8^th^ June 2015 (last patient last visit). Patients were recruited in the local community with advertisements and referrals by paediatricians. This study was performed according to the international guideline for clinical trials with pediculicides [12].

### Inclusion and Exclusion Criteria

Patients (>1 year) were included after a confirmed head lice infestation. All diagnoses were made by trained personnel at the clinical facility, using a designated plastic head lice comb (Elimax^®^, tooth gap 0.2 mm with rigid teeth). First, the hair was detangled in 4 parts. Next, every part of the hair was combed six times, starting at the scalp and ending at the hair tips. Head lice caught by the comb were left in the hair in order not to bias treatment. The severity of the head lice infestation was judged on a four-point severity scale: 0 = no relevant infestation (0–4 lice and/or nymphs present), 1 = mild (5–9 lice and/or nymphs present), 2 = moderate (10–24 lice and/or nymphs present) and 3 = severe (≥ 25 lice and/or nymphs present).

Only patients with at least five living lice or nymphs and five apparently living eggs present at the first visit were included. Exclusion criteria were: known sensitivity towards product ingredients, use of a pediculicide 4 weeks prior to study start, secondary scalp infection, chronic scalp disease, pregnancy or lactation and prior participation during the previous 4 weeks in any other clinical trial.

### Informed consent, Randomization, and Baseline Data

Study participants or their legal representative in case of minors gave written consent and the children also provided written or verbal assent, according to age, witnessed by the parent or guardian. Eligible subjects were randomly assigned to receive one of the two head lice treatments by a computer-generated code using balanced blocks of 6.

Baseline demographic data were collected on gender, age, hair length, and previous treatments. Patients agreed to be available for the 10-day duration of the study prior to enrolment.

### Blinding

Because of discernible differences in quality (e.g. smell, consistency, colour…) and in the administration process between both investigational products, patients and clinic personnel were able to recognize the products. In contrast, all subsequent assessments were performed by blinded study staff to avoid any product bias

### Study medication, dosage and administration

The test head lice shampoo was supplied in 100 ml plastic bottles by Oystershell Laboratories (Ghent, Belgium). The active ingredient is light, white mineral oil (pharmacopoeia-grade), approved for use in cosmetics and pharmaceutical products (concentration: 69.3%). Other ingredients are surfactants (amine laureth sulfate, laureth-4, and cocamide DEA; concentration range: 29–30%) to allow direct wash out, and perfume (<1%). The test shampoo is classified as a medical device as it exerts its effects through a physical mode of action (suffocation).

The control product, Goldgeist^®^ Forte solution, is a pediculicide containing pyrethrum extract (0.3%) and piperonyl butoxide (0.7%), which is a synergist of different insecticides, including pyrethroids. Pyrethrum has been used for centuries as an insecticide and lice remedy. It is made from dried flower heads, primarily of *Chrysanthemum cinerariifolium*. The main active ingredients are pyrethrins, but also cinerins and jasmolins. These agents kill head lice by acting on nerve cell membranes and interrupting muscle neurotransmission [3]. Goldgeist^®^ Forte solution was purchased from the market as 100-ml glass bottles (Eduard Gerlach GmbH, Pf. 1249; D- 32292 Lübbecke)

Patients received two treatments, a first application at visit 1 (day 0) and a second at visit 3 (day 7), as recommended by the German guidelines for head lice treatment, provided by the Robert Koch institute (central disease surveillance and prevention institute) [13], and according to the instructions for use of both products.

Application of the test head lice shampoo and the control product was performed as directed by the products’ leaflets. The amount of applied product depended on the length of the scalp hair, but no more was used than necessary to properly cover the scalp and hair. The exposure time did not exceed the prescribed 10 (+2) minutes for the test product and 45 (+5) minutes for the control product. Following application, the test shampoo was directly washed out (one rinse). The comparator product was rinsed away using only lukewarm water. No commercial shampoo was applied to exclude any potential effect on head lice.

### Efficacy evaluation

Infestation degree in both treatment groups was diagnosed using a plastic head lice comb, as described in section “Inclusion and exclusion criteria”. Assessments were made at the following time points: day 0 (visit 1), 24h post treatment (visit 2), day 7 (prior to second treatment; visit 3), and day 10 (visit 4).

The primary objective of this study was to demonstrate a cure rate of the test product being better than 70% for all baseline infestations at day 10 (visit 4). The cure rate is the proportion of patients without any living lice, corrected for re-infestation. The latter was defined as a) no adult lice or third stage nymphs present following the first treatment, and b) no more than two adult lice or third stage nymphs found by combing on day 10 [14].

The secondary efficacy objective was to show non-inferiority of the test product (assuming an outcome of 70% at day 10 for the medicinal control product). We refer to section “Statistical analyses” for more detailed information.

### Sample size

The pre-defined limit of 70% approximates the highest cure rate, reported in a recent clinical study with a comparable head lice product [15] and was assumed to be the minimal acceptable cure rate. Sample size calculation was performed using previous clinical data for the Silcap test product (not published). A sample size of 42 was required for a one sample chi-square-test comparing cure rate of 90% with a fixed limit of 70% (two sided test; alpha-level of 0.05; power = 90%; software nQuery advisor 7.0). For the control group, the identical sample size was used.

Assuming 10% drop-outs and a 5% rate for re-infestation, the sample size was multiplied by 1.1 x 1.05, leading to approximately 50 cases per group. Therefore, 107 patients were randomized, 53 for the test group and 54 for the control arm, respectively.

### Safety evaluation

As secondary objectives, safety and tolerability as well as acceptance of test head lice shampoo and the control product were evaluated. On day 0 and 7 (before and one hour after application), and at the final examination at day 10, local tolerability (subjective symptoms for burning, paraesthesia, pruritus) was evaluated. Study staff assessed skin (secondary infection, erythema, excoriation) and eye irritation on days 0, 1, 7 and 10. In addition, satisfaction with the esthetical effects was reported on day 0 and on day 7 after application. Global tolerability (“very good”, “good”, “moderate”, and “poor”) was evaluated by study staff and patients at the final examination at day 10.

### Statistical analyses

The statistical analysis complied with the International Conference on Harmonization (ICH) E9 Note for Guidance on Statistical Principles for Clinical Trials [16]. An independent statistical consultant performed analyses on the primary efficacy data set. The latter included all patients who received two treatments (50 in each arm at day 10) and one additional patient, who withdrew consent due to treatment failure in the test group (total, n = 101; test group, n = 50; control group, n = 50).

The cure rate (p) corresponds to the proportion of patients who were cured at day 10 among all patients that received any treatment at day 0. The aim of this study was to show superiority for the cure rate of the test product (pT) versus a pre-defined limit of 70%. This limit refers to cure rates, found for several formulations which are accepted for the intended use.

The following null-hypothesis was tested: H_0, prim_: p_T_ = 70%. If p_T_ > 70% and the null-hypothesis was rejected by a two-sided, one sample χ^2^-test at 0.05 level, superiority could be concluded. The control product was tested in the same way.

Regarding difference in cure rates of test and control product p_T_−p_R_, the following hypothesis was tested: H_0; sup_: p_T_−p_R_ = 0.

In case superiority could not be proven, non-inferiority would be tested. According to ICH guideline “Points to consider on switching between superiority and non-inferiority” [17], it is allowed to assess non–inferiority in to the absence of superiority. Prerequisites include a) predefinition of a non-inferiority margin, and b) no correction in terms of multiplicity. As non-inferiority margin (δ), a 7.5% worse cure rate was regarded as clinically not relevant. The following null hypothesis (tested α-level of 0.025) was used: H_0, NI_: pT–pR < δ, whereby δ = -7.5%. The lower, one sided 97.5% confidence interval of difference pT-pR was used for the test. If H_0, NI_ was rejected, non-inferiority could be shown.

All analyses were performed using Statistical Analysis System software, version 9.2 (SAS Institute Inc. Cary, NC, USA).

For better understanding the clinical results, post hoc analyses were performed. In this additional evaluation, only per protocol treated patients, who received two treatments (n = 50 in each arm), were included. This data is known as the “secondary efficacy set”. The absolute difference in cure rate, with two-sided 95% confidence interval, derived from the normal approximation to the binomial distribution, was calculated to compare the cure rates of both treatments.

Chi-square test for independence was used to compare the cure rate after correction for re-infestation on day 10. These analyses were performed with R version 3.1.1. (R-Core Team, 2015).

## Results

**Fig 1:**
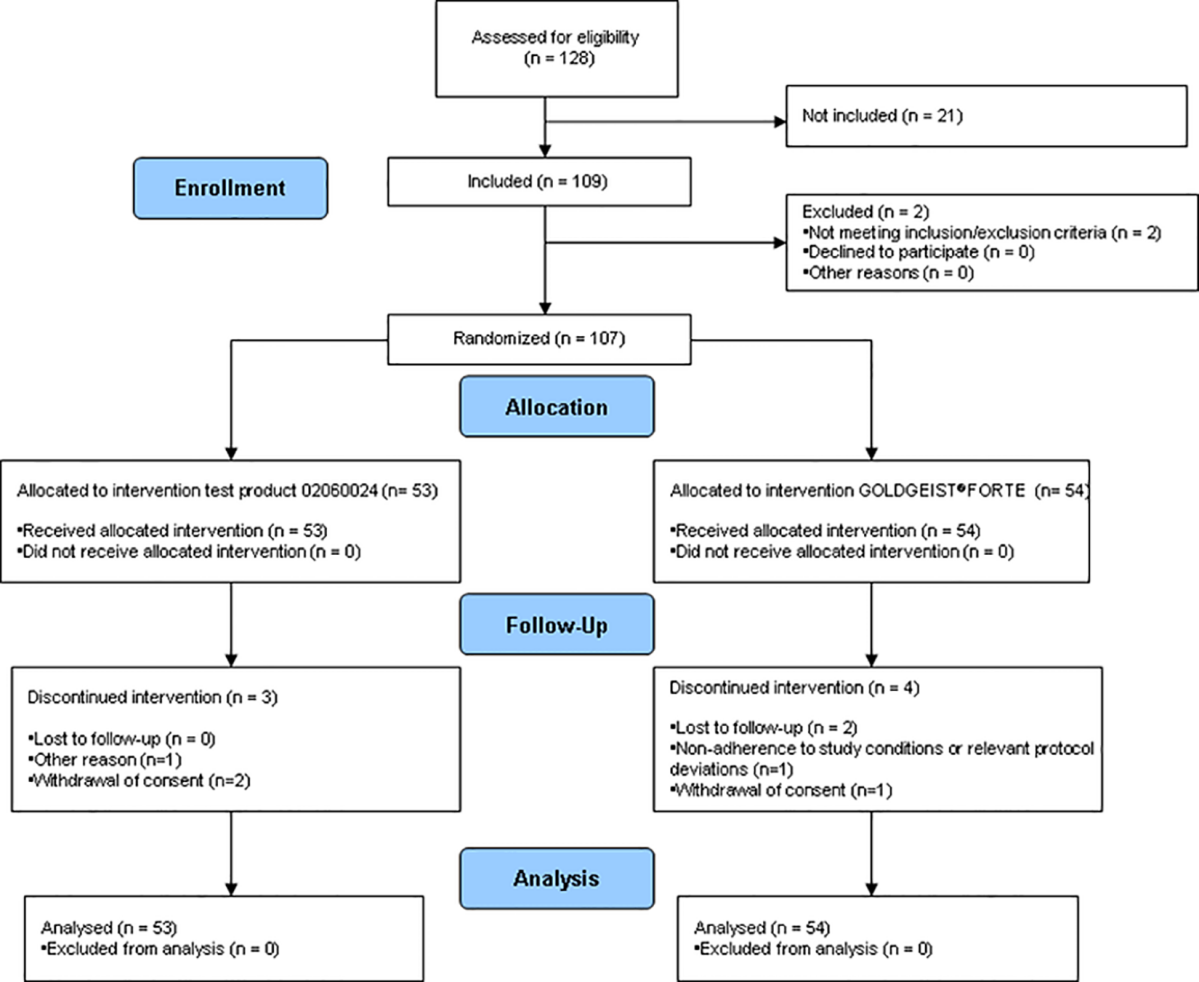
Disposition of study subjects.

Study data were collected between October 13^th^ 2014 and June 8^th^ 2015. Of the 109 subjects with acute head lice infestation (at least 5 living lice and 5 apparently living eggs) who gave informed consent, 107 were randomised and received first study treatment (n = 53 test product; n = 54 control product). Due to 7 drop-outs (test group, n = 3; control group, n = 4), 50 patients in each arm finished the study (Fig 1).

One of these drop-outs (test group) withdrew consent because of non-effective treatment. For this reason, this patient has been classified as not cured at visit 4 and was included into the cure rate analysis of visit 4. In total, data from 101 patients (test group n = 51, control group n = 50) were included in the “primary efficacy data set”.

### Baseline patient characteristics

In the test group, 44 female and 9 male subjects were included and in the control group 41 female and 13 male subjects. The average age in the study was 12.8 ± 9.0 years (mean ± standard deviation), ranging from 3 to 44.8 years. Except for three Asian subjects, all patients were Caucasian.

Fifty-eight patients (test group, n = 24, control group, n = 34) were classified as having a “mild”, 31 patients (test group, n = 20, control group, n = 11) having a “moderate”, and 18 patients (test group n = 9, control group, n = 9) having a “severe” infestation.

Before treatment (visit 0), the average number of adult live lice was 2.7 ± 4.1, ranging from 0 to 24. The average number differed slightly between the treatment arms (2.3 for test group versus 3.1 for the control group). The average number and distribution of nymphs per stage (I, II, III) did not considerably differ in both treatment arms, though the maximal number of nymphs in the test group was remarkably higher (46 versus 10).

In the secondary efficacy set, the number of live lice (adults and nymphs) ranged from 5 to 44 (median 8), and from 5 to 68 (median 10) in the test and control product group, respectively.

More patients with shoulder length hair were treated with the test product (20 versus 14), while more patients of the control group had short (15 versus 12) or mid-back long hair (25 versus 21). Summary of baseline characteristics is presented in Table 1.

**Table 1:** Demographics and baseline characteristics.

	Test head lice shampoo	Goldgeist® Forte	Total
**Age group**			
2 to < 6 years, *n* (%)	5 (9.4)	5 (9.3)	10 (9.3)
6 to < 11 years, *n* (%)	27 (50.9)	23 (42.6)	50 (46.7)
11 to < 18 years, *n* (%)	15 (28.3)	15 (27.8)	30 (28.0)
≥ 18 years, *n* (%)	6 (11.3)	11 (20.4)	17 (15.9)
**Race**			
White, *n* (%)	52 (98.1)	52 (96.3)	104 (97.2)
Asian, *n* (%)	1 (1.9)	2 (3.7)	3 (2.8)
**Sex**			
Male, *n* (%)	9 (17.0)	13 (24.1)	22 (20.6)
Female, *n* (%)	44 (83.0)	41 (75.9)	85 (79.4)
**Hair length**			
Short hair, *n* (%)	12 (22.6)	15 (27.8)	27 (25.2)
Shoulder length, *n* (%)	20 (37.7)	14 (25.9)	34 (31.8)
Mid back, *n* (%)	21 (39.6)	25 (46.3)	46 (43.0)
**Severity of head lice infestation**			
Mild (5–9 lice and/or nymphs present), *n* (%)	24 (45.3)	34 (63.0)	58 (54.2)
Moderate (10–24 lice and/or nymphs present), *n* (%)	20 (37.7)	11 (20.4)	31 (29.0)
Severe (more than 24 lice and/or nymphs present), *n* (%)	9 (17.0)	9 (16.7)	18 (16.8)
**Total**, *n* (%)	53 (100)	54 (100)	

### Efficacy results

For the test product arm, only 3 of 50 patients were infested at day 10. Two of them were assessed as re-infestation. Inclusion of the subject who discontinued the study due to treatment failure, yielded a cure rate (corrected for re-infestation) of 96.1%.

For the control group, 5 patients were not lice-free at day 10. Two of them were classified as re-infestation, yielding a cure rate (corrected for re-infestation) of 94%.

These data demonstrate that the primary objective (cure rate > 70%) was achieved for both products (p-value < 0.001).

Cure rates were already very high in both groups. With a difference of 2.1% (95% CI: -6.39%; 10.55%), the higher cure rate for the test shampoo did not translate into statistically significant superiority. However, non-inferiority of the test product vs. control was statistically established, taking into account a pre-defined margin of -7.5%.

Post hoc analyses, performed on the secondary efficacy set (100 patients, two treatments) demonstrated a cure rate of 98% and 94% for the test product and the control, respectively.

#### Number of living lice and proportion of lice-free subjects at each visit

Post hoc analyses showed that for all visits, cure rates were consistently higher for the test product when compared to the control. Data are summarized in Table 2, representing for each visit the number of lice-free subjects, also expressed as %.

**Table 2:** Cure rates at day 1 (Visit 2), day 7 (Visit 3), and day 10 (Visit 4).

Lice free	Test head lice shampoo (n = 50)		Goldgeist® Forte (n = 50)	
	N	%	N	%
Day 0	NA	NA	NA	NA
Day 1	45	90	40	80
Day 7	45	90	33	66
Day 10	47	94	45	90

Briefly, 24 hours after the first treatment, exactly 90% (45/50) of the subjects in the test group were lice free, whereas 80% (40/50) was cured in the control group. Prior to second treatment at day 7 (V3), there was a significant cure rate advantage of 24% for the test head lice shampoo: 90% of the subjects in the test product were lice-free vs 66% of the subjects in the control group (95% CI: 8.5%; 39.5%; p = 0.004).

After the first treatment, the number of subjects who remained lice-free until the end of the observation period was remarkably higher in the test head lice shampoo arm (78%; 39/50; 95% CI: 64.8%-87.2%) when compared to the control product arm (60%; 30/50; 95% CI: 46.2%-72.4%), showing a difference of 18% (95% CI: 0.2%; 35.8%; p<0.05).

**Fig 2:**
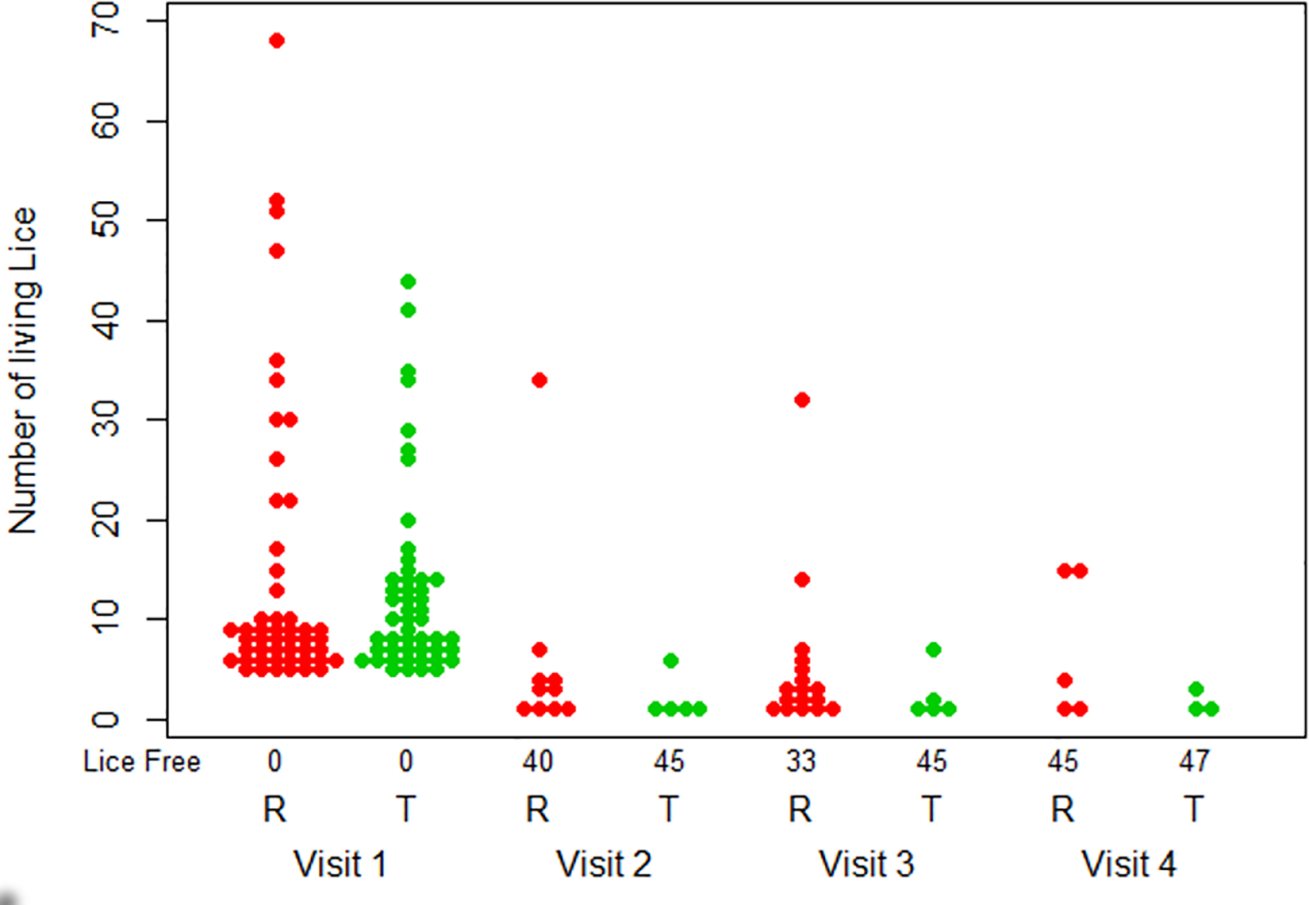
Number of lice per subject and subjects free of lice at each visit by treatment group (R: Control product; T: test head lice shampoo).

Moreover, the maximum number of live lice in uncured subjects was lower in the test product group than in the control group (Table 3 and Fig 2). This was the case for all visits: V2: 6 versus 34, V3: 7 versus 32, and V4: 3 versus 15 for the test group and control group, respectively. However, these higher scores were within the confidence intervals of both products.

**Table 3:** Number of live lice at day 0 (Visit 1), at day 1 (Visit 2), day 7 (Visit 3), and day 10 (Visit 4).

Number of live lice	Test head lice shampoo (n = 50)			Goldgeist® Forte (n = 50)		
	Min	Median	Max	Min	Median	Max
Day 0	5	8	44	5	10	68
Day 1	0	0	6	0	0	34
Day 7	0	0	7	0	0	32
Day 10	0	0	3	0	0	15

#### Satisfaction with esthetical effect

In general, the most subjects were satisfied with the esthetical effects following application. The easy wash out of the mineral oil product contributed to this appreciation. There were no remarkable differences in outcome between visit 1 and visit 3, with exception of “hair not too dry” (n = 6 vs. n = 2 for test product), and “pleasant scalp” (n = 14 vs. n = 9 for test product), both outcomes being in favour of the test product. The majority of the patients in both treatment arms were satisfied with the other esthetical effect after treatment (“scalp”, “normal greasiness of hair”, “good looking hair”, “fine shininess of hair”, and “fine volume of hair”).

### Safety evaluation

#### Local tolerability

Local tolerability was assessed over all visits (6 questionings), evaluating the following parameters: burning, paraesthesia, and pruritus. Observations were classified as mild, moderate, and severe. Data are summarized in Table 4.

**Table 4:** Summary of local tolerability parameters.

	Test head lice shampoo		Goldgeist® Forte	
Parameter	Number of subjects	Number of observations	Number of subjects	Number of observations
**Mild burning**	11	13	16	24
**Moderate burning**	1	1	2	2
**Severe burning**	0	0	0	0
**Mild paraesthesia**	22	32	17	32
**Moderate paraesthesia**	5	8	4	5
**Severe paraesthesia**	3	3	1	1
**Mild pruritus**	36	84	36	81
**Moderate pruritus**	19	21	16	22
**Severe pruritus**	5	9	4	4

Briefly, the number of “mild burning” observations was almost twice as high in the control group when compared to the test group. Moderate burning was only reported in the control arm, whereas severe burning was not reported in both groups.

“Mild paraesthesia” observations were identical in both arms, whereas only a limited number of “moderate and “severe paraesthesia” observations were made.

Assessment of pruritus demonstrated an equal number of mild and moderate cases for both groups. Finally, almost no subjects reported “severe pruritus” in both arms.

These data demonstrate good local tolerability for both products.

#### Global tolerability

All patients and study staff rated global tolerability as “good” or “very good”.

#### Skin irritations

For both investigational products, good skin tolerability was reported. Over all visits, no secondary infection was reported. Observations of “mild erythema” were twice as high in the control group (n = 8) than the test group (n = 4). The same observation was made for “mild excoriation” (test group, n = 3; control group, n = 8). One “moderate excoriation” was reported in the test group.

#### Eye irritations

For both investigational products, eye tolerability was good. Over all visits, no redness of the eyes was reported for the control product. Only a few cases of “mild redness” of left eye (N = 4) and right eye (N = 4) in the test group were reported.

#### Adverse Events and Serious Adverse Events

Only 18 adverse events (AEs) were reported (test group n = 11; control group n = 7) and were completely resolved until the end of study. No patient dropped out due to an AE.

Only 8 of them were drug related. In the test group “mild headache” (n = 1), “mild pruritus” (n = 1), “mild erythema” (n = 2) and “moderate administration site rash” (n = 1) were reported. For the control group, 2 patients reported a “mild headache” and one patient a “mild administration site erythema”. All other 10 AEs were not drug-related.

In summary, both products are well-tolerated, with a low number of AEs. Skin irritation and burning was higher in the control group, whereas some subjects reported eye irritation in the test group.

## Discussion

Treatment of head lice infestations remains a clinical relevant problem because of increasing resistance and safety concerns, observed with classic insecticidal pediculicides [3–7]. For this reason, alternative treatments have taken over the market in Europe during the last years, including silicone-based head lice products with a physical mode of action. For some of these products, both *in vitro* and clinical studies have confirmed their mode of action, efficacy and safety [15, 18–20].

For over a century, mineral oils have been widely applied as external insecticidal with the aim to cover and suffocate insects [8, 9]. However, limited clinical data is available on mineral oil-based pediculicides. Some manufacturers claim that mineral oil is equally effective as silicones by disrupting water and gas exchange via the respiratory spiracles in a similar manner [8–11]. The present study has been set-up to compare efficacy and safety of a mineral oil head lice shampoo (Mosquito Med Läuse Shampoo 10 in Germany; Silcap or Paranix shampoo in other countries) with a pyrethroid-based, reimbursed medicinal product (Goldgeist Forte^®^ solution).

All study procedures were standardised and performed in accordance with the clinical trial protocol and the GCP regulations. Only minor deviations from the clinical trial protocol occurred and none was judged clinically relevant. No subject was excluded from the analysis due to protocol deviations. For the results obtained from the present study, no inconsistencies between related measures were observed.

Two data sets were statistically and independently analysed. From the 107 randomized subjects, 100 completely finished the study and received two treatments. Each arm included 50 subjects. These data have been used for the post hoc analyses. However, the primary data set consisted of 101 patients. One additional patient in the test group, who withdrew consent because of treatment failure during the study, was included for cure rate analysis at day 4.

Regarding the primary objective of this study, statistical analyses demonstrated that treatment with the test product yields very high cure rates: 96.1% (primary data set) and 98% (secondary data set), respectively, at day 10 for all baseline infestations. The observed values were higher than the pre-defined limit of 70% (*p* < 0.001). The latter value approximates the highest cure rate (67.7%), reported in a recent clinical study with a comparable head lice product [15]. Nevertheless, in general, lower cure rates have been observed with pyrethroid-based treatments [3, 15, 21–24].

For the control product, the cure rate at day 10 for all baseline infestations was not significantly different when compared to the test product (primary and secondary data set: 94%). In other words, superiority could not be shown by a reliable margin. However, according to the ICH [17], it is allowed to assess non–inferiority in addition to superiority if a non-inferiority margin was predefined (-7.5% in the present study), and no corrections were made in terms of multiplicity. Taking into account a difference of 2.1% in cure rate between both treatment groups, non-inferiority was established.

Post hoc analysis revealed that over all visits, cure rates were higher for the test product than for the control, with a significant difference (95% CI: 8.5%; 39.5%; *p* = 0.004) at day 7 prior to treatment (test group, 90%; control: 66%). Day 7 was chosen to perform the second treatment, both based on the German guidelines for head lice treatment [13] and the instructions for use of both products.

Interestingly, a single application of the test product was sufficient to keep 78% (95% CI: 64.8%-87.2%) of all patients lice-free until the end of the observation period, being significantly (p<0.05) different from the control group (60%; 95% CI: 46.2%-72.4).

Moreover, the maximum number of live lice in uncured subjects was lower in the test product group when compared to the control group (all visits). However, these higher values lie within the confidence interval of both products.

Additionally, it is important to mention that a) the overall infestation degree was lower in the control group, and b) the fact that more patients in the control group received larger volumes of product as recommended (n = 8 versus 3 for control and test group, respectively).

The observed cure rates were higher than typically reported in the literature [3, 15, 21–24]. Diagnostic combing might have theoretically biased clinical outcome. However, in both groups, diagnosis was performed in an identical manner by experienced staff in such a way that no lice and eggs were damaged or lost. For the same reason, wet combing following treatment was not intended. Therefore, even if combing would have had an impact on final clinical outcome, the effect would be minimal and comparable in both groups.

Another explanation for the observed results may be the rigorous compliance with the clinical procedures that characterized this particular study population. A third reason we can offer for the high cure rate is the limited spread of pyrethrin resistance in the German population. Germany is an exception in Europe because it has a tradition of prescription control for pyrethroid and organophosphorus pediculicides on the market. We speculate that the study population in this particular region may have been less exposed to insecticidal treatments. For this reason, there may have been less selection for resistance when compared to other countries. In Germany, pyrethroid-based products are still present on the positive list of the Robert Koch Institute (the central disease surveillance and prevention institute), and are still broadly recommended for head lice control.

Results of ovicidal testing (at the end of the study) would have added extra value to the clinical data. However, the clinical test facility was not able to do such tests. These assays needed to be performed elsewhere but transport of the samples would have stressed the nits and thus negatively bias hatching results. However, previous *in vitro* tests demonstrated reproducible mortality rates in line with the observed clinical outcome (unpublished data).

With respect to safety, the tested mineral oil shampoo consists of ingredients which are approved for use in pharmaceutical and cosmetic products. On a regular basis, ingredient safety are assessed by expert panels (e.g. Cosmetic Ingredient Review expert, Scientific Committee on Consumer Safety…) and public health instances. Also, notification and auditing responsibilities for medical devices require a competent level of quality, safety and efficacy control. Present clinical safety data confirm results of different *in vivo* tests, performed according to specific ISO guidelines, to assess product safety. These test results are part of the medical device’s technical file and are essential for product approval by the responsible authorities (data not shown).

As treatment time is very short and the number of treatments to cure patients is low (1 or 2), the risk of side effects is very low. Indeed, the present study confirms that the oil-based shampoo is well-tolerated, with a low number of adverse events. Finally, both short treatment period (10 min) and high efficacy minimize the exposure risk. Similar observations were made for the comparator. Skin irritation and burning was higher in the control group, whereas a limited number of subjects reported eye irritation in the test product group. Nevertheless, there is raising concern regarding potential acute and long-term effects on human health, associated to the use of pyrethroids [7]. The higher treatment time (45 min) implies a higher exposure risk, which may in turn have an impact on long-term effects of pyrethroids.

Finally, the majority of the patients in both treatment arms were satisfied with the esthetical effects after treatment, including wash-out of the oily shampoo.

In conclusion, when compared to a pyrethroid-based product, the mineral oil shampoo may be a preferred option for head lice treatment. The ease of use (short exposure time), high efficacy, confirmed product safety, good esthetical effects, affordability and low risk for development of resistance are reassuring product characteristics that could improve treatment compliance and epidemiological control

## Supporting Information

S1 CONSORT Checklist Consort 2010 Checklist. (PDF)

S1 Protocol Study protocol. (PDF)
