# Supplementary material for: Efficacy and Safety of a Mineral Oil-Based Head Lice Shampoo: A Randomized, Controlled, Investigator-Blinded, Comparative Study
Source: PLoS One. 2016 Jun 10;11(6):e0156853. doi: 10.1371/journal.pone.0156853 (PMC4902190; doi:10.1371/journal.pone.0156853)
Supplement: S1 Protocol — (PDF) [file pone.0156853.s002.pdf]

1 Title Page

Study Protocol - Clinical Study: Phase IV

**Study Code Sponsor:** **NCLIN0091** <br>**Study Code CRO:** **14ct/mp37li**	**Study Title:** **A Randomised, Controlled, Investigator-Blinded, Comparative Study to Evaluate the Safety and Efficacy of a Head Lice Shampoo**

Working Title: [redacted] in Head Lice

**Version:** V03	**Date:** 2014-08-25

EudraCT No: 2014-002918-23

**Principal Investigator:**	Dr. Dörte Wolf<br>CardioSec Clinical Research GmbH<br>Peterstr. 5<br>D-99084 Erfurt<br>Tel: +49-(0) 361-789 197 40<br>Fax: + 49-(0) 361-789 197 44<br>E-Mail: doerte.wolf@cardiosec.de
**Sponsor:**	Oystershell Laboratories<br>Booiebos 24<br>B-9031 Drongen<br>Tel: + 32-(9) 377 248 5<br>Fax: + 32-(9) 377 968 0

Confidential, Property of CardioSec Clinical Research GmbH

2 Synopsis

Study phase	IV	
Test product (Medical Device) and route of administration	Test:	[redacted] locally applied
Reference product (Drug), and route of administration	Reference:	GOLDGEIST® FORTE, locally applied
Study Code Sponsor	NCLIN0091	
Study Code CRO	14ct/mp37li	
EudraCT No.	2014-002918-23	
Indication studied	Head lice infestation	
Study objectives	Evaluation of safety and efficacy of product [redacted] for the treatment of head lice in comparison to reference product GOLDGEIST® FORTE:<br>• Assessment of the efficacy of treatments for head lice<br>• Assessment of local tolerability<br>• Assessment of global tolerability by the patient<br>• Assessment of global tolerability by the investigator<br>• Assessment of skin irritation<br>• Assessment of eyes irritation<br>• Assessment of satisfaction with the esthetical effect<br>• Characterisation of safety and tolerability of the investigational product considering Adverse Events in the study population	
Regulatory	In this study a drug-product (GOLDGEIST® FORTE) and a medical device (the test product [redacted] are involved. According to EU and German regulations the rules for clinical trials with drug-products (AMG, GCP-V etc.) and the rules for clinical trials with medical devices (MPG, MPKPV, MPV etc.) have to be followed. Before the trial can start, approval according to drug-regulation and medical-device regulation has to be granted.<br>Medical-device regulations allow the competent authorities to exempt certain, non-critical medical-devices from the general requirement of approval of the trial. In this trial the authorities were asked to exempt the test-product from the approval process concerning the medical device.<br>Nevertheless approval according to drug-regulations is required. For this reason, the terms used in this protocol are closer to those used in the area of drug-regulation than those used in the medical-device area.	
Population	100 patients with head lice of both gender, aged ≥ 1 year	
Study design	Monocentric, randomised, controlled, investigator-blind study to evaluate safety and efficacy of the investigational products	
Duration of treatment	Repeated treatment of test product [redacted] or GOLDGEIST® FORTE on study day 0 and study day 7,<br>The duration of treatment for the test product [redacted] will be 10 minutes. A deviation from plus 2 minutes will be accepted. For the reference product a treatment time from at least 30 minutes up to 45 minutes is given. A deviation up to plus 5 minutes will be accepted.	
Total dose per patients	Total dose per patient depends on hair length. No more of the investigational product will be used, as necessary to properly cover the hair and scalp.<br>For product [redacted]<br>The hair and scalp should be covered completely.<br><br>For GOLDGEIST® FORTE:<br>Short hair (< than to the shoulders): about 25 mL,	

	Hair with shoulder length: about 35-40 mL,<br>Mid-back hair (> than to the shoulders until mid-back) about 75 mL will be used.<br>Infants will be treated with a maximum amount of 25 mL.
In- / Exclusion criteria	**Inclusion criteria**<br>To be eligible, the following inclusion criteria must be met:<br>1. Gender: male/female<br>2. Age: ≥ 1 year of age at the time of signing the informed consent<br>3. Patients with active head lice infestation of at least 5 live lice and 5 apparently live eggs<br>4. Patient or his/her guardian must be capable of understanding and providing written informed consent<br>5. The patient or his/her legal representative must give written informed consent, after having been oral and written informed about benefits and potential risks of the trial, as well as details of the insurance taken out to cover the subjects participating in the study<br>6. Patients must agree to not use any other ant-lice treatment for the duration of the study<br>7. Female patients: are women of childbearing potential who test negative for pregnancy and agree to use a reliable method of birth control or remain abstinent during the study. Methods of contraception considered acceptable include oral contraceptives, contraceptive patch, intrauterine device, vaginal ring, diaphragm with contraceptive gel, or condom with contraceptive gel<br>-or<br>are women of non–childbearing potential, defined as: women who have had surgical sterilization (hysterectomy, bilateral oophorectomy, or tubal ligation),<br>-or<br>women who are ≥60 years of age.<br><br>**Exclusion criteria**<br>A participant is not eligible, if any of the following exclusion criteria are present:<br>1. Known allergic reactions or hypersensitivity to the active ingredients used or the constituents<br>2. Patients with known skin allergies, multiple drug allergies or multiple allergies to cosmetic products<br>3. Pregnant or breast feeding women<br>4. Patient underwent treatment with any form of head lice treatment within the last 30 days prior to the Screening Visit (day 0)<br>5. Patients with chronic scalp disorder<br>6. Individuals on systemic or topical drugs or medications, including systemic antibiotics, which in the opinion of the investigative personnel may interfere with the study results<br>7. Subjects with hair longer than mid-back<br>8. Patients suspected or known not to follow instructions<br>9. Patients or his/her legal representative who are unable to understand the written and verbal instructions, in particular regarding the risks and inconveniences they will be exposed to as a result of their participation in the study<br>10. Previous participation in this study or participation in any other investigational trial within the preceding 30 days

	11. The patients are investigator site personnel directly affiliated with this study and/or their immediate families. Immediate family is defined as a spouse, parent, child, or sibling, whether biological or legally adopted<br>12. The patients are Oystershell employees or are employees of third-party organizations involved in the study.
Analytical methodology	Not applicable
Number of study centres	1 site in Germany
Total number of patients	100 patients enrolled, drop outs will be replaced
Baseline characteristics	• Demographic data<br>• Case history incl. medical history<br>• Confirmation and severity of head lice infestation<br>• Skin irritation baseline-assessment pre-treatment (secondary infection, erythema, excoriation)<br>• Eyes irritation baseline-assessment pre-treatment
Primary objective	1. The primary objective is to show that the cure rate after local application of test product [redacted] is better than a predefined limit. It has to be shown, that the test product [redacted] achieves a cure rate superior to 70% (cure rate at the end of day 10, corrected for re-infestation).
Secondary objective	**<u>Efficacy</u>**<br>2. To show that the cure rate at the end of day 10 (corrected for re-infestation) for the reference product GOLDGEIST® FORTE for all baseline infestations is better than 70%.<br>3. To show that the test product [redacted] has a cure rate superior to that of the reference product GOLDGEIST® FORTE<br>4. In case that superiority cannot be shown: to show that the test product [redacted] is non-inferior to reference product GOLDGEIST® FORTE regarding cure rate. Thereby a non-inferiority margin of 7.5% will be used.<br>5. Efficacy of the investigational products for head lice for mild and moderate baseline infestations (cure rate at the end of the study day 10, corrected for re-infestation)<br>6. Efficacy of the investigational products for head lice for all baseline infestation (cure rate at day 1)<br>7. Efficacy of the investigational products for head lice for mild and moderate baseline infestations (cure rate at day 1)<br>**<u>Safety</u>**<br>8. To evaluate local tolerability by patient: subjective symptoms (burning, paraesthesia, pruritus): 0h, 1h, 24h, 7d and 10d p.a. (descriptive evaluation)<br>9. To evaluate global tolerability by the patient and study staff (number of subjects with global tolerability ratings of “very good”, “good”, “moderate”, “poor”, descriptive evaluation) at 10d after application<br>10. To evaluate skin irritation by study staff (secondary infection, erythema, excoriation) on day 0, 1, 7 and 10<br>11. To evaluate eye irritation by study staff on day 0, 1, 7 and 10<br>12. To evaluate characterisation of safety and tolerability of the investigational products considering Adverse Events in the study population
Efficacy variables	• Cure rate at the end of day 10 (corrected for re-infestation) for the test product [redacted] for all baseline infestations (primary parameter)

	• Cure rate at the end of day 10 (corrected for re-infestation) for the reference product GOLDGEIST® FORTE for all baseline infestations (secondary parameter)<br>• Difference of cure rates (secondary parameter)<br>• Cure rate at day 10 (corrected for re-infestation) for mild and moderate baseline infestations, (secondary parameter)<br>• Cure rate at day 1 for the test product [redacted] for all baseline infestations (secondary parameter)<br>• Cure rate at day 1 for mild and moderate baseline infestations, (secondary parameter)
Safety variables	• Local tolerability: subjective symptoms (burning, paraesthesia, pruritus): 0d (0h, 1h), 1d, 7d (0h, 1h) and 10d p.a. by patient (secondary parameter)<br>• Global tolerability assessment at 10d by patient and study staff (secondary parameter)<br>• Skin irritation assessment: 0d (0h, 1h), 1d, 7d (pre-treatment, 0h, 1h) and 10d p.a. (secondary infection, erythema, excoriation) by study staff (secondary parameter)<br>• Eyes irritation assessment: 0d (0h, 1h),1d, 7d (pre-treatment, 0h, 1h) and 10d p.a. by study staff (secondary parameter)<br>• Questioning on general well-being and Adverse Events (AEs): 0d (0h, 1h), 1d, 7d (0h, 1h) and 10d p.a. (secondary parameter)
Patient recorded outcomes	• Questioning on the satisfaction with the esthetical effect of the anti-lice products by patient: day 0 and day 7 after treatment and hair drying (secondary parameter)
Pharmacokinetic variables	Not applicable
Statistical methods	Descriptive statistics of all endpoint variables will be tabulated (continuously scaled variables: mean, standard deviation, median, min, max, categorically scaled variables: counts and percentages) by visit and treatment.<br>**Populations**<br>Safety population (SAF): All enrolled subjects who administered the investigational product, independently of the duration of treatment.<br>Per-protocol population (PP): All enrolled subjects, who show no major protocol deviations (to be defined before database lock). All subjects who provide valuable data.<br>**Hypotheses**<br>**The primary aim** of the study is to show superiority for the cure rate of investigated treatment p_T versus a predefined limit of 70%.<br>The following null hypothesis will be tested:<br>$H_{0,prim}$: $p_T = 70\%$<br>If $p_T > 70\%$ and the null hypothesis is rejected by a two sided, one sample χ^2-test at 0.05 level, superiority will be concluded.<br>**Secondary objectives:**<br>The reference product will be tested in the same way.<br>Regarding difference of cure rates of test and reference product p_T-p_R, the superiority of p_T vs p_R will be tested by following null hypothesis:<br>$H_{0,sup}$: $p_T - p_R = 0$.<br>According ICH guideline “Points to consider on switching between superiority and non-inferiority” it is allowed to assess non–inferiority in addition to superiority if superiority cannot be shown. A non-inferiority margin has to be predefined. Moreover, no correction in terms of multiplicity is necessary. As non-inferiority margin δ, a 7.5% worse cure

	rate will be regarded as clinically not relevant.<br>The following null-hypothesis will be tested on α-level of 0.025.<br>$H_{0,NI}$: p_T - p_R<δ, whereby δ = -7.5%<br>The lower, one sided 97.5% confidence interval of difference p_T-p_R will be used for the test. If $H_{0,NI}$ will be rejected, non-inferiority will be shown.<br>The difference of cure rates p_T-p_R as well the cure rates p_T and p_R will be presented with two sided 95% confidence interval.<br>The other objectives will be investigated exploratory by descriptive statistics as well as by appropriate statistical tests.<br>Details analysis and will be fixed in a detailed Statistical Analysis Plan (SAP).
Planned start of study	September 2014

3 Flow chart

**Procedure**	**Screening Visit V01**			**Evaluation Visit V02**	**Second Treatment Visit V03**	**Follow-up Visit V04**
Study day	Day 0			Day 1 (±3h)	Day 7 (+1d)	Day 10 (-1d; +2d)
	-2h	0h	+1h	24h after end of administration (±3h)		
Informed consent process	•					
Case history incl. medical history	•					
Demographic (ethnic group, gender, age, hair length, body weight, height etc.)	•					
Checks for concomitant medication	•			•	•	•
Physical examination of the scalp to verify head lice infestation by study staff	•					
Pregnancy test in urine[A]	•					
Inclusion/Exclusion criteria check	•					
Randomisation		•				
Skin irritation (baseline-assessment, pre-treatment): secondary infection, erythema, excoriation by blinded study staff[B]		•			•	
Eyes irritation assessment (baseline-assessment, pre-treatment) by blinded study staff[B]		•			•	
Administration of the investigational product by un-blinded study staff[C]		•			•	
Global tolerability assessment by blinded study staff and patient						•
Local tolerability assessment (subjective symptoms: burning, paraesthesia, pruritus) by the patient [D]		•	•	•	•	•
Skin irritation assessment: secondary infection, erythema, excoriation by blinded study staff[D]		•	•	•	•	•
Hair scalp assessment for living lice (all stages) by blinded study staff				•	•	•
Eyes irritation assessment by blinded study staff[D]		•	•	•	•	•
Questioning on general well-being by blinded study staff		•	•	•	•	•

**Procedure**	**Screening Visit V01**			**Evaluation Visit V02**	**Second Treatment Visit V03**	**Follow-up Visit V04**
Study day	Day 0			Day 1 (±3h)	Day 7 (+1d)	Day 10 (-1d; +2d)
	-2h	0h	+1h	24h after end of administration (±3h)		
Questioning on AE by blinded study staff			●	●	●	●
Questioning on patient's combing				●	●	●
Check of restrictions				●	●	●
Questioning on satisfaction with the esthetical effect [E]			●		●	
Discharge from clinical study						●

A Pregnancy tests (urine) will be performed in all female patients with childbearing potential prior to study start

B Baseline assessments within 1h prior to administration on day 0 and day 7, on day 7 the pre-treatment assessment for irritations (skin, eyes) have to be used for decision about withdrawal due to severe irritation

C The time of exposure to the investigational products on the uncovered hair will not exceed 10 + 2 minutes for the test product [redacted] and 30 – 45 + 5 minutes for the reference product GOLDGEIST® FORTE

D Assessments after administration will be performed directly after start of the treatment plus 5 minutes as well as after the end of administration (the contact time is over and the investigational product is rinsed) within 15 minutes.

E Satisfaction with the esthetical effect will be performed on day 0 and day 7 after treatment and hair drying by the patients

For all other assessments a deviation up to ± 20 min from scheduled time will be accepted.

4 Signature pages

4.1 Signature Page I

The undersigned confirm that they agree to conduct the study under the conditions described in this protocol.

I agree to comply with all relevant SOPs required for the conduct of this study. I further agree to ensure that all associates assisting in the conduct of study are informed regarding their obligations. I further agree to ensure that all associates assisting in the conduct of study are informed regarding their obligations.

Digitally signed by Dorien Staljanssens
DN: cn=Dorien Staljanssens, o=Oystershell laboratories, ou=R&D, email=ds@oystershell.com, c=BE
Date: 2014.08.25 14:57:48 +02'00'

Date:

Dorien Staljanssens
Project Manager, Sponsor
Oystershell Laboratories

(DD/MM/YYYY)

4.2 Signature Page II

The undersigned confirm that they agree to conduct the study under the conditions described in this protocol. I agree to comply with all relevant SOPs required for the conduct of this study. I further agree to ensure that all associates assisting in the conduct of study are informed regarding their obligations. I will provide copies of the protocol and all information available on the investigational medicinal product relating to pre-clinical and prior clinical experience, which was furnished to me by the Sponsor, to all physicians, nurses and other personnel who participate in this study and will discuss this material with them to assure they are fully informed regarding the investigational medicinal product and the conduct of the study.

I finally agree that this study will be reviewed by an Ethical Committee in accordance with the Declaration of Helsinki 2013, and I agree to conduct this study in full accordance with the ICH guideline for Good Clinical Practice and the appropriate law.

D. Wolf

Dr. med. Dörte Wolf
Medical Director, Principal Investigator
CardioSec Clinical Research GmbH

Date: 25.08.2014
(DD/MM/YYYY)

4.3 Signature Page III

The undersigned confirm that they agree to conduct the study under the conditions described in this protocol.

I agree to comply with all relevant SOPs required for the conduct of this study. I further agree to ensure that all associates assisting in the conduct of study are informed regarding their obligations. I further agree to ensure that all associates assisting in the conduct of study are informed regarding their obligations.

Date: 26/AUG/2014

Dr. rer. nat. Thomas Keller
Trial Statistician
ACOMED statistik

(DD/MM/YYYY)

5 Table of contents

1 Title Page ... 1
2 Synopsis ... 2
3 Flow chart ... 7
4 Signature pages ... 9
4.1 Signature Page I ... 9
4.2 Signature Page II ... 10
4.3 Signature Page III ... 11
5 Table of contents ... 12
6 Abbreviations ... 15
7 Responsibilities and contact addresses ... 16
8 Introduction ... 18
8.1 Properties of the investigational products ... 19
8.2 Pharmacokinetics ... 21
9 Study Rationale ... 21
10 Study objectives ... 23
10.1 Primary objective ... 23
10.2 Secondary objectives ... 23
11 Study design ... 24
12 Investigational products ... 25
12.1 Selection of doses in the study ... 25
12.2 Identity of the investigational products ... 25
12.3 Packaging and labelling ... 26
12.4 Drug accountability ... 26
13 Justification of the study ... 27
13.1 Risks related to the participation of the clinical trial ... 27
13.1.1 Procedure related risks ... 27
13.1.2 Risks related to the investigational drugs ... 27
13.1.2.1 Test product [redacted] ... 27
13.1.2.2 GOLDGEIST® FORTE ... 27
13.1.3 Precautionary measures ... 28
13.1.4 Risk/benefit evaluation ... 28
13.1.5 Assessment of risk and burden ... 29
13.1.5.1 Risk assessment ... 29
13.1.5.2 Burden assessment ... 31
13.1.6 Stop criteria in case of elevating risk/burden ... 33
14 Study population ... 33
14.1 Inclusion criteria ... 33
14.2 Exclusion criteria ... 34

14.3 Method of assigning volunteers to treatment groups 35
14.4 Blinding 35
14.5 Removal of volunteers from treatment or assessment 35
15 Clinical performance of the study 36
15.1 Definition of study periods 36
15.2 Study Visits and measures 37
15.2.1 Screening Visit V01 (day 0): study initiation, registration, enrolment, randomisation, first administration 37
15.2.2 Evaluation Visit V02 (day 1): 24h p.a. 38
15.2.3 Second Treatment Visit V03 (day 7) 39
15.2.4 Follow-up Visit V04 (day 10) 39
15.3 Restrictions 40
15.3.1 Miscellaneous 40
15.4 Administration of investigational products 40
15.4.1 Subject and treatment compliance 41
15.5 Assessments 41
15.5.1 Global tolerability assessment 41
15.5.2 Local tolerability assessment 42
15.5.3 Skin irritation assessment 42
15.5.4 Eyes irritation assessment 42
15.5.5 Assessment of esthetical properties 43
15.5.6 Assessment of the hair and scalp (diagnostic combing) 44
15.6 Efficacy measurements 44
15.6.1 Re-infestation 44
15.7 Safety measurements 44
15.7.1 Specific safety measurements 44
15.7.2 Checks for general well-being 45
15.7.3 Pre-treatment signs and symptoms and Adverse Events 45
15.7.3.1 Definition of pre-treatment signs and symptoms and Adverse Events 45
15.7.3.2 AE-Assessment 46
15.7.3.3 Documentation and Reporting 48
Follow-up of subjects after Adverse Events 49
15.8 Prior and concomitant therapy 50
15.8.1 Concomitant treatment in the event of relevant AEs 50
15.9 Appropriateness of measurements 50
15.9.1 Clinical laboratory parameters 50
15.9.2 Clinical parameters 51
15.10 Handling and documentation of clinical data 51
15.11 Clinical data management 53

16 Biostatistics 53
16.1 Populations for analyses 53
16.2 Description 53
16.3 Analysis of primary objective 53
16.4 Analysis of secondary objectives 54
16.5 Determination of sample size 54
16.6 Further statistical analyses 55
16.7 Statistical software 55
16.8 Quality control for statistical procedures 55
17 Evaluation of efficacy parameters 55
18 Evaluation of safety parameter 55
19 Evaluation patient reported outcomes 56
20 Quality assurance 56
21 Ethics 57
21.1 Independent Ethics Committee (IEC) 57
21.2 Ethical conduct of the study 57
21.3 Volunteer information and consent 57
21.4 Insurance 58
22 Minors enrolled in the study 58
22.1 Informed consent and assent 58
22.1.1 Infants and pre-school children 59
22.1.2 Children of school age (from about 6 years old) 59
22.1.3 Consent and assent in adolescents 59
22.2 Informed consent (and assent for children) of families with different cultural background 59
22.3 Facilities and staff involved 60
23 Initiation of the trial 60
24 Protocol Amendments 60
25 Reporting 60
26 Record keeping 60
27 Publication policy 60
28 Reference list 61

6 Abbreviations

ADR	Adverse Drug Reaction
AE	Adverse Event
ANOVA	Analysis of Variances
BfArM	Federal Institute for Drugs and Medical Devices (German Drug Agency)
BMI	Body-Mass Index
C	Concentration
CAS	CAS Registry Number, CAS = Chemical Abstracts Service
CPMP	Committee for Proprietary Medicinal Products
CRF	Case Report Form
CRO	Contract Research Organisation
CV	Coefficient of Variation
EU	European Union
GCP	Good Clinical Practice
GLP	Good Laboratory Practice
GMP	Good Manufacture Practice
g	Gramm
ICH	International Conference on Harmonisation of Technical Requirements for Registration of Pharmaceuticals for Human Use
IEC	Independent Ethics Committee
IFRA	International Fragrance Association
IMP	Investigational Medicinal Product
l/L	Litre
max	Maximum
min	Minimum
mg	Milligram
mL	Milliliter
p.a.	post administrationem
PDF	Portable Document Format
PTSS	Pre-treatment Sign and Symptom
QAU	Quality Assurance Unit
QC	Quality Control
SAE	Serious Adverse Event
SD	Standard Deviation
SOP	Standard Operating Procedure
TT	Text Table
TMF	Trial Master File

7 Responsibilities and contact addresses

Responsibility	Name	Address
Sponsor	Oystershell Laboratories	Booiebos 24, B-9031 Drongen<br>Tel: + 32-(9) 377 248 5<br>Fax: + 32-(9) 377 968 0
Project Manager Sponsor	Dorien Staljanssens	**Oystershell Laboratories**<br>Booiebos 24, B-9031 Drongen<br>Tel: + 32-(9) 262 047 8<br>Fax: + 32-(9) 377 968 0<br>E-Mail:<br>dorien.staljanssens@oystershell.com
Principal Investigator	Dr. Dörte Wolf	**CardioSec Clinical Research GmbH**<br>Peterstr. 5, D-99084 Erfurt<br>Tel: +49-(0) 361-789 19 740<br>Fax: +49-(0) 361-789 19 744<br>E-Mail: doerte.wolf@cardiosec.de
Co-Investigators	Dr. Jörg Kremser<br>Holger Sörgel<br>Dr. Carina Schenk	**CardioSec Clinical Research GmbH**<br>Peterstr. 5, D-99084 Erfurt<br>Tel: +49-(0) 361-789 19 740<br>Fax: +49-(0) 361-789 19 744<br>E-Mail: joerg.kremser@cardiosec.de<br>E-Mail: holger.soergel@cardiosec.de<br>E-Mail: carina.schenk@cardiosec.de
Project Manager CRO	Dr. Anja Wildner	**CardioSec Clinical Research GmbH**<br>Peterstr. 5, D-99084 Erfurt<br>Tel: +49-(0) 361-789 19 740<br>Fax: +49-(0) 361-789 19 744<br>E-Mail: anja.wildner@cardiosec.de
Clinical Study Site	CardioSec Clinical Research GmbH	Peterstr. 5, D-99084 Erfurt<br>Tel: +49-(0) 361-789 19 740<br>Fax: +49-(0) 361-789 19 744
Data Management	Monika Huschak	**CardioSec Clinical Research GmbH**<br>Peterstr. 5, D-99084 Erfurt<br>Tel: +49-(0) 361-789 19 740<br>Fax: +49-(0) 361-789 19 744<br>E-Mail: monika.huschak@cardiosec.de
Packaging of investigational product<br>[redacted]	Oystershell Laboratories	Booiebos 24, B-9031 Drongen<br>Tel: + 32-(9) 377 248 5<br>Fax: + 32-(9) 377 968 0
Statistics	Dr. Thomas Keller	**ACOMED Statistik**<br>Fockestr. 57, D-04275 Leipzig<br>Tel: +49-(0) 341 391 019 5<br>Fax: +49-(0) 341 391 019 6<br>E-Mail: info@acomed-statistik.de

Pharmacovigilance	Isabelle Geeraert	Booiebos 24, B-9031 Drongen<br>Tel: + 32-(9) 377 248 5<br>Fax: + 32-(9) 377 968 0<br>E-Mail: regulatory@oystershell.be
Monitoring	Dorien Staljanssens	**Oystershell Laboratories**<br>Booiebos 24, B-9031 Drongen<br>Tel: + 32-(9) 262 047 8<br>Fax: + 32-(9) 377 968 0<br>E-Mail:<br>dorien.staljanssens@oystershell.com

8 Introduction

Head lice (*Pediculus humanus capitis*) are parasitic insects which infest the head and neck of humans and attach their eggs to the base of the hair shaft. They are common, particularly school-aged children. Head lice are tiny (2-3 mm in length), wingless insects which live close to the human scalp and feeding exclusively on human blood. They cannot fly, jump or swim and are spread by direct contact with an infested individual and move by crawling. Close contact between individuals are more likely routes of infestation than shared bed lines, combs or hair brushes, hats, towels. Head-to-head contact is the most common route of lice transmission.
A head lice infestation is not the result of dirty hair or poor hygiene. Head lice can affect all types of hair, irrespective of its condition and length. Head lice do not carry disease and only affects humans. They cannot be passed on to animals or be caught from them. Without a host head lice usually die within 24 hours.

A female head louse lays up to 3-4 eggs per day and cementing them to the hair shaft (often close to the roots) with glue where they are kept warm by the scalp. The eggs are about 0.8 mm in length and take on average 1-2 weeks to hatch. After hatching, the louse nymph leaves behind the egg shell (also called nit) and takes 7-10 days to mature. Head lice can survive on the human host for up to 30 days. All stages are blood-feeders and bite the skin four to five times daily to feed. They inject substances to keep the blood from coagulating. Head lice are mostly found in the nape of the neck and the area behind the ears.
In most cases, itching is the main symptom of head lice infestation caused by the coagulative substances or the crawling of the lice over the scalp.

Head lice are very common. About 6-12 million infestations, mainly children, occur in the United States each year. In Germany there are 600-1000 new infections per 100.000 children each year. It occurs more frequently in girls than boys. It occurs less frequently in African Americans than other races; this is thought to be due to the shape and thickness of the African American hair shaft.
Diagnosis is made by the discovery of live louse/lice on the head of an individual. It is not always easy to see head lice because of the small size and colour (grey/brown, sometimes colourless). Generally, the person looking for the live lice and/or nits should use a magnifying lamp and a detection comb. The hair should be divided into small sections to look for moving lice and nits. The areas around the top of the ears and at the neck line are the most common locations for nits.
Treatment is recommended if several nits or lice are found on an individual's scalp.

The "Bundesamt für Verbraucherschutz und Lebensmittelsicherheit" has published a notice of tested and recognized compositions and methods for controlling animal pests. The following medical devices and drugs are registered: Jacutin® Pedicul Spray (allethrin I), InfectoPedicul® (permethrin), GOLDGEIST® FORTE (pyrethrum), mosquito® Läuse-Shampoo, NYDA®, Jacutin® Pedicul Fluid.

The development of resistance is increasing so alternative treatments are being used more frequently. All pediculicides recommend the use of a nit comb following their application to remove any residual nits from the hair shafts.

The use of alternative products without chemical pediculicides is also increasing due to the development of resistance with traditional medications. Many of these alternatives work by a physical mode of action (e.g. suffocation or dehydration), rather than a chemical action, so

resistance is unlikely to develop. The investigational product, which is used in this study, makes use of this physical mode of action.

This study is designed to compare the effectiveness and safety of product [redacted] versus GOLDGEIST® FORTE following two applications.

8.1 Properties of the investigational products

<u>Test product:</u> [redacted]

The test product [redacted] is a medical device for the treatment of head lice infestation. The active component is white mineral oil which has been used safely in a wide range of cosmetic products (even baby products), medications, soaps, pesticides, lubricants.

The test product [redacted] is a yellow, translucent solution consisting of mineral oil, [redacted]

This combination makes the formula foam on the hair after the addition of water. This allows the mineral oil to be washed out without the use of an additional shampoo. The formula contains [redacted]

The product formulation of test product [redacted] is qualitatively and quantitatively identical with the marketed product Silcap lice shampoo (Oystershell Laboratories).

<u>Mineral Oil:</u> white mineral oil (CAS 8042-47-5)

White mineral oil is no hazardous substance or mixture according to Regulation (EC) No. 1272/2008. This substance is not classified as dangerous according to Directive 67/548/EEC.

White mineral oil is a mixture of liquid hydrocarbons, essentially paraffinic and naphthenic in nature obtained from petroleum. It shows no acute oral toxicity no skin or eye irritating, was not sensitising and has no genetic toxicity or carcinogenity [1].

[redacted]

[redacted]

[redacted]

[redacted]

[redacted]

[redacted]

[redacted]

[redacted]

The following mode of action is described: The oil acts as a suffocant and blocks the respiratory spiracles of the louse. Due to the oils' high affinity for the epicuticle of the louse it is also likely that it solubilises the protective waxes making the louse more vulnerable to dehydration. The mineral oil in test product [redacted] constitutes the active component responsible for killing head lice and eggs. It is tailored to the hydrocarbon composition of the epicuticle of head lice so as to obtain a maximum affinity for it. After application of the product on the hair, the mineral oil quickly engulfs the head lice and kills them. The activity is based on two principles: the mineral oil suffocates the insect as well as dehydrates it.

Reference product: GOLDGEIST® FORTE

The active ingredient of GOLDGEIST® FORTE, the reference product in this study, is pyrethrum extract. Pyrethrum has been used for centuries as an insecticide and as a lice remedy. This natural insecticide is made from dried flower heads, primarily of *Chrysanthemum cinerariifolium.* The main active ingredients are pyrethrins, also cinerins and jasmolins. Pyrethroids are synthetic, or man-made, versions of pyrethrins, one common example is permethrin. Pyrethrin is usually combined or synergized with piperonyl butoxide. Both chemicals act synergistically to kill lice by acting on nerve cell membranes and interrupting signal travelling between brain and muscles. The lice become paralyzed and die because they are unable to breath.

Pyrethrum has been extensively studied for its effects on people and the environment. Like all insecticides, pyrethrum is used to have a toxic effect on insects. Pyrethrum has a very good toxicity profile. [7]. For mammals, doses that elicit toxic reactions are significantly larger than the exposures people typically experience in using pyrethrum based products.

On broken skin, pyrethrum produces irritation and sensitisation, which is further aggravated by sun exposure. Absorption of pyrethrum through the stomach and intestines and through the skin is slow [8]. However, humans can absorb pyrethrum more quickly through the lungs during respiration. Pyrethrum (as 100%) has an acute oral toxicity in rats of LD_{50} 3500 mg/kg and an acute skin toxicity in rabbits of LD_{50} 19000 mg/kg [9]. Piperonyl butoxide (as 100%) has an acute oral toxicity in rats of LD_{50} 6150 mg/kg and an acute skin toxicity in rabbits of LD_{50} 1880 mg/kg.

Response appears to depend on the pyrethrum compound used. Inhaling high levels of pyrethrum may bring about asthmatic breathing, sneezing, nasal stuffiness, headache, nausea, lack of coordination, tremors, convulsions, facial flushing and swelling, and burning and itching

sensations [10]. The lowest lethal oral dose of pyrethrum is 750 mg/kg for children and 1000 mg/kg for adults [9]. At high doses, pyrethrum can be damaging to the central nervous system and the immune system [11].

Pyrethrins and their metabolites are not known to be stored in the body nor excreted in the milk. The urine and faeces of people given oral doses of pyrethrum contain chrysanthemumic acid and other metabolites [12]. In mammals, tissue accumulation has not been recorded.

Overall, pyrethrins have low chronic toxicity to humans and the most common problems in humans have resulted from the allergenic properties of pyrethrum. Pyrethrum can produce skin irritation, itching, pricking sensations and local burning sensations [9].

Pyrethrins appear to have low reproductive toxicity [13].

8.2 Pharmacokinetics

A topical exposure with white mineral oil showed no evidence of any hazard at any dose in multiple species, which is supported by the long and uneventful human use of white mineral oil in drug and non-drug topically applied products [14]. The absorption of mineral oil from the gastrointestinal tract is limited.

Pharmacokinetic data in humans is available: A commercial formulation containing 0.3% C-pyrethrins was applied to the skin of 6 human volunteers at a rate of 5.5 mg pyrethrins/cm. The unabsorbed material was washed off 30 minutes later. It was calculated that 2% of the dose was absorbed (range 0.06 to 4.1%). Following oral dosing of rats with 50 or 100 mg/kg body weight C-pyrethrin I, peak blood concentrations were found 5 to 6 hours after dosing in male and 6 to 8 hours after dosing in females. Most radioactivity was excreted during the first 72 hours. In males, 32 to 47% of the dose was recovered from urine and 53 to 71% from faeces. The corresponding values for females were 50 to 57% and 50 to 52%. Several metabolites were identified in urine. The major metabolite in urine samples was chrysanthemum dicarboxylic acid [15].

Pyrethrin demonstrates very low dermal absorption and is quickly metabolized to inactive metabolites which are excreted in the urine. Because absorption after topical application is very limited, occasional pyrethrins and piperonyl butoxide use is acceptable in nursing mothers [16].

9 Study Rationale

In this study, a drug-product (GOLDGEIST® FORTE) and a medical device (the test product [redacted] are involved. According to EU and German regulations the rules for clinical trials with drug-products (AMG, GCP-V etc.) and the rules for clinical trials with medical devices (MPG, MPKPV, MPV etc.) have to be followed. Before the trial can start, approval according to drug-regulation and medical-device regulation has to be granted.

Medical-device regulations allow the competent authorities to exempt certain, non-critical medical-devices from the general requirement of approval of the trial. In this trial the authorities were asked to exempt the test-product from the approval process concerning the medical device.

Nevertheless approval according to drug-regulations is required. For this reason, the terms used in this protocol are closer to those used in the area of drug-regulation than those used in the medical-device area.

Infestations with head lice are ubiquitous and affect children, and to lesser extent adults, in both developed and developing countries [17]. With an estimated incidence of approximately 1.500 new cases per 10.000 children per year (Germany) head lice infestation is one of the most frequent infectious diseases in children aged eight to twelve years [18]. The infestation causes considerable discomfort, as intensive itching, especially during night rest, thereby leading to sleep disturbances.

There are different treatment possibilities. Topical application of pediculicide agents, such as permethrin, allethrin and phenotrin, is one possible therapy for head lice treatment in patients with pediculosis capitis. Alternative products without chemical pediculicides are another option. Many of these alternatives work by a physical mode of action (e.g. suffocation or dehydration), rather than a chemical action, so resistance is unlikely to develop. The investigational product used in this study makes use of this physical mode of action.

The sponsor Oystershell has developed a lice shampoo Silcap[redacted]which is already on the European market. In addition, Oystershell is going to license the same formulation under a different market brand to a third party. This licensed product [redacted] is the test product in this study. The preclinical evaluation was performed on the older product Silcap [redacted] Nevertheless, since composition and administration of [redacted] and [redacted] are identical, reference in the study protocol is made to the documentation of Silcap [redacted] [19].

For both products [redacted] and [redacted] declarations of conformity have been established, both products belong to the medical device class I, according to annex IX of the medical devices directive.

The purpose of the study is to show the efficacy and safety of test product [redacted]against head lice in comparison to a licenced product as precondition for the reimbursement by the statutory health fund.

The reference product GOLDGEIST® FORTE is a licenced and recommended anti-head lice agent from the “Bundesamt für Verbraucherschutz und Lebensmittelsicherheit” in Germany, therefore the sponsor decided to use this as reference product.

The study will be performed in patients ≥1 year of both genders with confirmed diagnosis of head lice infestation.

For safety documentation, the descriptive evaluation of the local tolerability assessment using the 4 point score for subjective symptoms, the documentation of adverse events, a global tolerability assessment by the subject and the study staff, an assessment of the eyes and skin irritation will be used.

Reference is made to the following guidelines:

- Declaration of Helsinki, Version 2013
- Note for Guidance on Good Clinical Practice (CPMP/ICH/135/95)
- GMP regulation, Annex 13, July 2003
- German Medical Products Law (MPG), August 2013
- Ordinance on Clinical Trials with Medical Devices (MPKPV), May 2010

- German Institute of Medical Documentation and Information (DIMDI) Ordinance, May 2010
- Medical Devices Regulation (MPV), May 2010
- Medical Devices Directive (MDD) (93/42/EWG)
- Note for Guidance on Clinical Safety Data Management: Definitions and Standards for Expedited Reporting (CPMP/ICH/377/95)
- German Drug Law (AMG), October 2012
- GCP Regulation (GCP-Verordnung), October 2012
- Federal Directives applicable to the Medical Corps (Berufsordnung für die deutschen Ärztinnen und Ärzte), Version 2011
- ICH E9 Note for Guidance on Statistical Principles for Clinical Trials (CPMP/ICH/363/96, 1998)
- Guideline on Clinical Requirements for Locally Applied, Locally Acting Products, Containing Know Constituents (CPMP/EWP/239/95)
- Guideline on the Choice of the Non-inferiority Margin (CPMP/EWP/2158/99)
- Points to Consider on Switching between Superiority and Non-inferiority (CPMP/EWP/482/99)

10 Study objectives

The aims of this study are the evaluation of efficacy and safety of test product [redacted] in comparison to GOLDGEIST® FORTE for the treatment of head lice.

10.1 Primary objective

The primary objective is to show that the cure rate after local application of test product [redacted] is better than a predefined limit. It has to be shown, that the test product [redacted] achieves a cure rate superior to 70% (cure rate at the end of day 10, corrected for re-infestation).

<u>Hereby, the following primary endpoint is investigated:</u>

1. Cure rate at the end of day 10 (corrected for re-infestation) for the test product [redacted] for all baseline infestations

10.2 Secondary objectives

Secondary objectives refer to comparison of the cure rate of test product [redacted] with those of reference product GOLDGEIST® FORTE using the following endpoints:

Secondary endpoint: Difference of cure rates (as defined as primary endpoints).

Secondary efficacy objectives:

2. To show that the cure rate at the end of day 10 (corrected for re-infestation) for the reference product GOLDGEIST® FORTE for all baseline infestations is better than 70%
3. To show that the test product [redacted] has a cure rate superior to that of the reference product GOLDGEIST® FORTE

4. In case that superiority cannot be shown: to show that the test product [redacted] is non-inferior to reference product GOLDGEIST® FORTE regarding cure rate. Thereby a non-inferiority margin of 7.5% will be used.

Further secondary objectives refer to objectives 3-4, however evaluated in subgroups or other time points.

5. Efficacy of the investigational products for head lice for mild and moderate baseline infestations (cure rate at the end of the study day 10, corrected for re-infestation)
6. Efficacy of the investigational products for head lice for all baseline infestation (cure rate at day 1)
7. Efficacy of the investigational products for head lice for mild and moderate baseline infestations (cure rate at day 1)

As further secondary objectives, safety and tolerability of test product [redacted] or reference product GOLDGEIST® FORTE as well as the acceptance of the investigational products will be evaluated.

Secondary safety objectives:

8. To evaluate local tolerability by patient: subjective symptoms (burning, paraesthesia, pruritus): 0h, 1h, 24h, 7d and 10d p.a. (descriptive evaluation)
9. To evaluate global tolerability by the patient and study staff (number of subjects with global tolerability ratings of "very good", "good", "moderate", "poor", descriptive evaluation) at 10d after application
10. To evaluate skin irritation by study staff (secondary infection, erythema, excoriation) on day 0, 1, 7 and 10
11. To evaluate eye irritation by study staff on day 0, 1, 7 and 10
12. To evaluate characterisation of safety and tolerability of the investigational products considering Adverse Events in the study population

11 Study design

This single-centre, randomised, controlled, investigator-blinded, comparative study will be conducted in 100 patients (female and male patients, ≥1 year of age) with confirmed diagnosis of head lice infestation. The patients will be randomised in a 1:1 ratio on both treatment arms.

In this study a repeated treatment, as recommended in literature [20] will be performed. For two-treatment protocols it is recommended that the second treatment should be applied on day 7 to day 9 and the final assessment should be on day 10.

Both investigational products will be tested as given in the product leaflets in compliance with the above mentioned treatment recommendations.

Patients complying with the in- and exclusion criteria will receive the study medication according to a patient number, allocated in ascending order in correspondence with their inclusion into the study.

Treatment will be performed as described in chapter 15.4 (Administration of the investigational products). The member of the study staff who will administrate the treatment will be un-blinded because administration procedure, smell, consistency and colour of pediculicides differ. In any case, the member of the study staff performing the subsequent assessments (assessor) will be blinded.

After informed consent procedure, confirmation of the diagnosis, check of exclusion and inclusion criteria and randomisation the first administration of the investigational products will be performed by the study staff. At the next visit (24 hours p.a.) patients will be re-inspected for living lice, as well as for safety assessments.

On study day 7, after re-inspection for living lice the second administration with the same investigational product as in Visit 1 will be performed by the un-blinded study staff.

On study day 10 the final visit will be performed at the study centre for final assessments of tolerability and efficacy, performing the procedures listed in the general trial flow chart (see chapter 3). In case of a treatment failure or a re-infestation the patient will be referred to his/her practitioner.

12 Investigational products

12.1 Selection of doses in the study

In this study the individual dose is chosen, according to the leaflet or the instruction of use, depending on the length of the scalp hair.

For treatment with test product [redacted] the hair and scalp should be covered completely with the product.

For the treatment with the reference product GOLDGEIST® FORTE patients with short hair will receive approximately 25 mL, while patients with hairs reaching the subject's shoulder will receive up to 35-40 mL. Patients with hairs from the subject's shoulder until the mid-back hair will be treated with about 75 mL of the investigational product. Infants will be treated with a maximum amount of 25 mL.

No more of the investigational products will be used, as necessary to properly cover the hair and scalp.

The time span of exposure to the investigational products on the uncovered hair will not exceed 10 (+ 2) minutes for the test product [redacted] and 30 – 45 (+ 5) minutes for the reference product GOLDGEIST® FORTE as recommended in the instructions for use.

The corresponding amount of the investigational products will be evenly dispensed in dry hair over its full length, with special diligence on the base of the hair near the scalp. The applied amount of both investigational products will be documented for each patient in the CRF.

12.2 Identity of the investigational products

The investigational test product [redacted] is supplied by Oystershell Laboratories. The following samples of the investigational products have been chosen for the study:

TT 1: Pharmaceutical information on investigational products

	Test	Reference
Product name	[redacted]	GOLDGEIST® FORTE
Manufacturer	Oystershell Laboratories	Eduard Gerlach GmbH
Formulation	Shampoo	Solution
Active ingredient	[redacted]	Pyrethrum extract 0.3 g/100 g<br>(Extract of *Chrysanthemum cinerariaefolium* blossoms (1:2,5), adjusted to 25 % pyrethrins with isoparaffin, extracting agent: 1) n-hexan 2) methanol/isoparaffin)
Packaging	100 mL	250 mL
Batch No.	NA	NA
Excipients	[redacted]	5-[2-(2-Butoxyethoxy) ethoxymethyle]-6-propyle-1,3-benzodioxole (Piperonylbutoxide)...........0.7 g/100 g<br>Chlorocresole...................0.9 g/100 g<br>Diethylenglycole.............40.0 g/100 g<br>Sodium-Laurylethersulfphate-Solution 27 %<br>Purified Water 2-Propanole, Caprylocaproyl Macrogolglycerides, Isoparaffin (C13-C14), Perfume, Quinolone yellow E 104, Phosphoric acid.
Licence No.	NA	6495154.00.00

12.3 Packaging and labelling

The investigational test product [redacted] will be provided, packed by and labelled by Oystershell Laboratories. The reference product GOLDGEIST® FORTE will be purchased from the market and will be used unchanged in the study.

All investigational products are to be stored in a locked area. Both investigational products have to be stored at room temperature; GOLDGEIST® FORTE has to be stored light protected.

12.4 Drug accountability

The use and fate of investigational products must be documented. After completion of the study, the containers including any remaining investigational product will be orderly destroyed by CardioSec GmbH.

A written explanation regarding the disposition of missing products or their containers is required.

13 Justification of the study

13.1 Risks related to the participation of the clinical trial

13.1.1 Procedure related risks

There is no procedure related risk identifiable.

13.1.2 Risks related to the investigational drugs

13.1.2.1 Test product [redacted]

The test product [redacted] has been marketed since 2009 in 40 countries under the brand name Silcap lice shampoo or Paranix. After treatment with Silcap shampoo the following adverse effects were reported in a previous clinical trial [21]:

- Mild transient burning sensation and redness of the face skin.
- Mild transient burning sensation on the eye after contact to the shampoo

Warnings and safety information according to the leaflet of the medical device:

Do not use in case of hypersensitivity to mineral oils. In the case of a rash or signs of irritation/allergy, stop the treatment immediately. Do not use this product when the scalp is already very irritated or injured.

Make sure you rinse out the product well. Always respect the duration of the treatment (10 minutes). Do not use the product under occlusion, e.g. by covering up the hair with a cap or wrapped foil.

Avoid any contact with the eyes and mucous membranes (mouth and nose). If the product comes into contact with the eyes, rinse out thoroughly with water.

For external use only. In case of accidental ingestion contact a doctor or the anti-poison Centre.

Keep out of the reach of children.

Keep treated hair away from open fire. Do not smoke during treatment.

White mineral oil is no hazardous substance or mixture according to Regulation (EC) No. 1272/2008. This substance is not classified as dangerous according to Directive 67/548/EEC.

13.1.2.2 GOLDGEIST® FORTE

The investigational product GOLDGEIST® FORTE contains pyrethrum extract as the active ingredient.

The following Adverse Events are described in package information leaflet which have been reported after treatment with GOLDGEIST® FORTE:

very frequently	≥ 1/10
frequently	≥ 1/100 and ≤ 1/10
occasionally	≥ 1/1000 and ≤ 1/100
rarely	≥ 1/10000 and ≤ 1/1000
very rarely	≤ 1/ 10000 and single cases

Skin:

Rarely: local skin irritations, temporary itching and redness of the treated skin areas

Central nervous system and sense organs:

Very rarely: contact sensitivity

Warnings and safety information's:

Chlorocresol may produce an allergic reaction.

Contact with eyes, mucous membranes, sore skin or lesions should be prevented, as this could cause itching, irritation or burning of the skin and eyes. In case of accidentally contact the affected area has to be carefully rinsed with sufficient amount of clear water.

Do not use when the scalp is already very irritated or injured.

As stated in the exclusion criteria, no patient with known allergic reactions to the active ingredients used or to constituents of the pharmaceutical preparation with both IMPs, as well as chrysanthemum and other composite plants, will be included in this study.

13.1.3 Precautionary measures

Special precautionary measures are not considered to be necessary for this study. In case of emergency, standard emergency procedures will be employed. The Principal Investigator is to be consulted and informed immediately.

The Principal Investigator will provide all the necessary emergency equipment and specially trained staff to handle emergency events during this study.

All cases of emergency (e.g. occurrence of SAE (=Serious Adverse Event) and/or occurrence of increasing numbers of AE's (= Adverse Event), which will result in an unacceptable risk-benefit ratio) will be immediately reported to the project manager of CardioSec Clinical Research GmbH.

13.1.4 Risk/benefit evaluation

Test product [redacted] as well as the comparator GOLDGEIST® FORTE will be administered according to the instructions for use by the study staff.

White mineral oil, the active ingredient of test product [redacted] is no hazardous substance or mixture and is not classified as dangerous according. It has been used safely in a wide range of products (e.g. cosmetics, baby products, medications, lubricants). The test product [redacted] is only suitable for head lice (not public lice) and is recommended for children from 1 year of age and older.

Pyrethrin, the active ingredient of GOLDGEIST® FORTE, are used against a broad range of pests including ants, aphids, roaches, fleas, flies, ticks and also lice. They are available in dusts, sprays, and aerosol "bombs" and they may be mixed with synthetic pesticides or other natural products. They break down quickly, have a short residual, and have low mammalian toxicity, making them among the safest insecticides in use. However, they might cause allergic skin

reactions in some people. Overall, pyrethrins have low chronic toxicity to humans and the most common problems in humans have resulted from the allergenic properties of pyrethrum.

The reference product GOLDGEIST® FORTE is suitable for head lice, public lice and clothes lice and there are no age restrictions for usage.

Adverse events following administration of product [redacted] or GOLDGEIST® FORTE will be monitored. Like in any trial, participating patients will be monitored during the assessment periods. No further special safety investigations will be carried out during the study as the investigational products known to be well tolerated.

The occurrence of severe drug related AE's is very unlikely after treatment with test [redacted] and GOLDGEIST® FORTE.

After consideration of the benefits and risks, the performance of the study is considered ethically justifiable, as the expected future therapeutic benefits of the study preparation appear to be greater than the risks to the volunteers.

13.1.5 Assessment of risk and burden

13.1.5.1 Risk assessment

Qualitatively and quantitatively the composition of the test product [redacted] is the same as that of Silcap lice shampoo which is already licensed in at least 40 countries for the treatment of head lice in children of 1 year and older.

GOLDGEIST® FORTE is already licensed for the treatment of head lice, pubic lice and clothes lice.

Furthermore, the treatments follows the recommendations on the instruction for use and will be applied by trained study staff, thus a decreased general risk in comparison to a home-based usage will be expected.

Due to the short treatment duration, the low concentrations of the active substances, its limited local administration and the minimal absorption of the drug even in irritated skin, there is only a low risk of systemic availability due to penetration. Thus systemic side effects are not expected, neither for adults nor infants.

Nevertheless all patients will be observed for signs of clinical toxicity during the entire study. Among others, checks for general well-being will be performed at 0 h (directly after application) and 1 h after application also as on day 1, 7 and 10 p.a.

Definition of risk- and burden-level

0	No or minimal risk/burden
1	Minor increase over minimal risk
2	Greater than minor increase over minimal risk

Minimal risk/burden is defined as "probability of harm or discomfort not greater than those ordinarily encountered in daily life or during the performance of routine physical or psychological examinations or tests".

Risk assessment

**Issue**	**IMP**	**Routine physical or psychological examinations, daily life, standard treatment**	**Treatment in clinical trial with IMP**	**Risk level (treatment with IMP)**
Adverse Events	Test product [redacted]	Only very rare or rare. (Only transient AEs were reported in a previous study, also in general side effects after local use of mineral oils are rare.)	No other side effects than those already known are expected to occur. In contrast the treatment will be performed by trained study staff, thus over dosage and risks by misapplication will be limited.<br>In case of allergic reactions immediate professional treatment will be provided, if necessary.	*0*
	Reference product GOLDGEIST® FORTE	Only very rare or rare. (Only transient AEs where reported in the leaflet of GOLDGEIST® FORTE predominantly involving the drug exposed parts of the skin.)	No other side effects than those already known are expected to occur. In contrast the treatment will be performed by trained study staff, thus over dosage and risks by misapplication will be limited.<br>In case of allergic reactions immediate professional treatment will be provided, if necessary.	*0*
Affection of the lungs	Test product [redacted]	Mineral oil aspirated into the lungs can cause pneumonia	Proper application of the IMP by trained staff will reduce direct contact with skin or aspiration into lungs.	*0*
	Reference product GOLDGEIST® FORTE	Pyrethrins can be absorbed through the lungs and skin.	Proper application of the IMP by trained staff will reduce direct contact with skin or aspiration into lungs.	*0*
Contact with eyes and mucous membranes (Violation against warnings and precautions)	Test product [redacted]	Product is for external use only, contact with eyes and mucous membranes should be avoided.	Application by trained study staff will be done, preventing contact of the IMP with the eyes and mucous membranes. In case of accidentally contact the affected area will be carefully rinsed with a sufficient amount of clear water.	*0*

Issue	IMP	Routine physical or psychological examinations, daily life, standard treatment	Treatment in clinical trial with IMP	Risk level (treatment with IMP)
	Reference product GOLDGEIST® FORTE	Product is for external use only, contact with eyes and mucous membranes should be avoided.	Application by trained study staff will be done, preventing contact of the IMP with the eyes and mucous membranes. In case of accidentally contact the affected area will be carefully rinsed with a sufficient amount of clear water.	*0*

13.1.5.2 Burden assessment

Therapy with pyrethrum containing products as well as mineral oil containing products is an effective and recommended treatment of head lice infestation in patients of early infancy as well as for adults.

Handling and application of both investigational products in this study will be the same as given in the leaflets.

The design of the protocol takes into account the objective target of minimizing risks, distress and discomfort. The study site is staffed with experienced personnel knowledgeable in dealing with the medical and psychosocial needs of children. CardioSec provides a comfortable setting with age-appropriate furniture, food, and play equipment. Minor burden due to the study design are listed below and the level of burden is assigned.

Burden assessment

Issue	Routine physical or psychological examinations, daily life, standard treatment	Treatment in clinical trial with IMP	Burden level (treatment with IMP)
Time of visits	Usually first visit at a paediatrician or practitioner for diagnosing and initiating treatment, subsequent follow up visits to verify treatment success (necessary to regain permission to attend e.g. the kindergarten)	Visit times are minimized to a maximum of about 3 hours on study day 0 (for informed consent process, determination of head lice infestation as well as treatment) and shorter visits on study day 1, 7 and 10 (final examination); individual attendance at the clinical ward will be timed according to the needs of the paediatric patients and/or infant patient‘s guardian, time of waiting will be no longer than 30 minutes, play equipment suitable for children for their entertaining will be provided.<br>Duration and extent of pre- and post- study examination does not differ significantly from common examinations necessarily done outside the clinical ward by a paediatrician or practitioner, when head lice infestation is diagnosed and treatment is indicated.	***0***
Dry combing for assessment of hair scalp for living lice	Standard procedure with fine toothed lice comb procedure	Not different from investigation by a paediatrician or practitioner outside the clinical ward.	***0***
Hair washing procedure	Comparable with daily life	Not different to daily life (could be performed by the infant’s guardian in case of very young patients, if necessary)	***0***
Application procedure and “time of taking effect”	Done according to the leaflet of the medical device recommended “time of taking effect”: 10 minutes	Application will be done as recommended in the leaflet of the medical device “time of taking effect” will be 10 minutes.	***0***
	Done according to the leaflet of GOLDGEIST® FORTE recommended “time of taking effect”: 30-45 minutes	Application will be done as recommended in the leaflet of GOLDGEIST® FORTE “time of taking effect” will be 45 minutes.	***0***
Assessments of tolerability and safety	N.A.	Requires just a few minutes of elevated concentration and time	***1***

The level of burden will be carefully monitored by the study staff throughout the entire study, especially during the in-house periods. Properly trained and experienced personnel knowledgeable and skilled in dealing with the paediatric population and its age-appropriate

needs, including skills in performing paediatric procedures ensures an adequate and equal treatment of all patients and minimizes any potential risk, distress or discomfort.

13.1.6 Stop criteria in case of elevating risk/burden

The level of risk and burden will continuously monitored as it may evolve over time, during the trial and with evolving knowledge.

The test product [redacted] and the reference product GOLDGEIST® FORTE will be applied twice (day 0 and day 7) during the study. In case of AEs of non-tolerable degree, the treatment will be stopped. The investigational products will then be washed off and applicable treated if necessary.

14 Study population

Hundred (100) non-institutionalised patients of both genders ≥1 year of age with a diagnosed head lice infestation will participate in the study.

The reference product [redacted] is not intended for children under 1 year of age. The reference product GOLDGEIST® FORTE could be used without any age restriction. Therefore, infants under 1 year of age will not be enrolled in the study.

According to the following guidelines women should principally include in clinical research.

- Detailed guidance on the application format to be submitted to ethic committees (Guidance document to the EC directive 2001/20/EC; April 2004): justification for the selection of trial subjects.
- **CIOMS/WHO International Ethical Guidelines**, Guideline 16

  Women as research subjects: “Investigators, sponsors, or ethical review committees should not exclude women of reproductive age from biomedical research. The potential for becoming pregnant during a study should not, in itself, be used as a reason for precluding or limiting participation….”

Both investigational products are intended for use in humans of both genders, therefore women should be included in the study, if this can be justified from a safety point.

The test product [redacted] is not foreseen for use by pregnant or breast-feeding women. For the reference product GOLDGEIST® FORTE there are no restrictions with respect to pregnant or breast-feeding women. Therefore, pregnant and lactating women will be not included into the study.

Special attention will be given to women with childbearing potential being enrolled in the study with respect to advice, examination and pregnancy testing. However women with childbearing potential will be requested to apply adequate contraceptive.

Patients will be enrolled in accordance with the following inclusion and exclusion criteria.

14.1 Inclusion criteria

To be eligible, the following inclusion criteria must be met:

1. Gender: male/female
2. Age: ≥ 1 year of age at the time of signing the informed consent
3. Patients with active head lice infestation of at least 5 live lice and 5 apparently live eggs

4. Patient or his/her guardian must be capable of understanding and providing written informed consent
5. The patient or his/her legal representative must give written informed consent, after having been oral and written informed about benefits and potential risks of the trial, as well as details of the insurance taken out to cover the subjects participating in the study
6. Patients must agree to not use any other ant-lice treatment for the duration of the study
7. Female patients:

   are women of childbearing potential who test negative for pregnancy and agree to use a reliable method of birth control or remain abstinent during the study. Methods of contraception considered acceptable include oral contraceptives, contraceptive patch, intrauterine device, vaginal ring, diaphragm with contraceptive gel, or condom with contraceptive gel

   -or

   are women of non–childbearing potential, defined as: women who have had surgical sterilization (hysterectomy, bilateral oophorectomy, or tubal ligation),

   -or

   women who are ≥60 years of age.

14.2 Exclusion criteria

A participant is not eligible, if any of the following exclusion criteria are present:

1. Known allergic reactions or hypersensitivity to the active ingredients used or the constituents
2. Patients with known skin allergies, multiple drug allergies or multiple allergies to cosmetic products
3. Pregnant or breast feeding women
4. Patient underwent treatment with any form of head lice treatment within the last 30 days prior to the Screening Visit (day 0)
5. Patients with chronic scalp disorder
6. Individuals on systemic or topical drugs or medications, including systemic antibiotics, which in the opinion of the investigative personnel may interfere with the study results
7. Subjects with hair longer than mid-back
8. Patients suspected or known not to follow instructions
9. Patients or his/her legal representative who are unable to understand the written and verbal instructions, in particular regarding the risks and inconveniences they will be exposed to as a result of their participation in the study
10. Previous participation in this study or participation in any other investigational trial within the preceding 30 days
11. The patients are investigator site personnel directly affiliated with this study and/or their immediate families. Immediate family is defined as a spouse, parent, child, or sibling, whether biological or legally adopted.
12. The patients are Oystershell employees or are employees of third-party organizations involved in the study.

The exclusion criteria are chosen to assure that patients with specific risks for administration of the investigated drug and patients with conditions that may have an impact on study parameters are excluded.

14.3 Method of assigning volunteers to treatment groups

This study will be randomised and investigator-blinded. When patients will arrive at the site they get a screening number. After the patient or his/her legal representative will give his/her written informed consent the patients will receive the subject number. Each patient who meets the inclusion and exclusion criteria will be assigned the lowest yet unassigned random number available at the centre. Randomisation will be performed during the Screening Visit V01 on day 0.

14.4 Blinding

As both products are substantially different from each other in terms of packaging, smell and application method, it is not possible to do a double-blinded trial. Both, the subjects and the clinic personnel that will apply the investigational products, will be able to recognize the products. Therefore, an investigator-blinded study will be performed, and an independent assessor (investigator or study staff performing the assessments of hair and scalp, eyes, as well the efficacy and safety evaluations) will be blinded to the treatment [20]. Appropriate measures will be taken to keep the assessor blind during the whole study. The assessor will not be involved in any procedure of the trial involving the handling, storage, and use of the products.

14.5 Removal of volunteers from treatment or assessment

Withdrawal criteria

- Serious Adverse Events (SAE's)
- Diseases requiring treatment that occur during the study, which do not constitute SAE's but which, in the opinion of the Principal Investigator, would probably prevent achievement of the study objectives.
- Non-adherence to the study conditions or relevant deviations from procedures as established in the study protocol.
- Withdrawal of consent.
- Duration of treatment < 10 minutes for [redacted] or < 30 minutes for GOLDGEIST® FORTE.
- Severe irritation of the skin or the eyes on pre-treatment assessment on day 7
- Use of any other anti-head lice therapy.

All details and reasons for removal of trial subjects from the study will be recorded in the study termination section of the CRF. CRFs of all trial subjects entered into the study have to be kept in the documentation of the study. Reasons for withdrawal of trial subjects will be entered into the final report of the study. Every effort has to be made to follow-up trial subjects who terminate with drug-related AE's in order to determine the final outcome.

Follow-up of withdrawn patients, drop outs

A drop out is a patient, who prematurely discontinues participation after being enrolled. If a patient drops out, possible PTSS (Pre-treatment Sign and Symptom) or AE's will be recorded as long as necessary. A post-study examination should be performed if possible.

All PTSS/AE's documented during the study for the drop out will be considered in the assessment. Other data recorded for the drop out are contained in the individual patient listing in an appendix to the study report.

Replacements

Patients who prematurely discontinue participation will be replaced.

Removal of volunteers from assessment

Screening failures

Screening failures are volunteers who were screened but not enrolled.

Screening failures including reason will be listed in the screening list. All data obtained within the screening will be documented in the patient's record. In case that entries were made to a CRF it will be archived, but not entered in the database.

Screening failures will not be listed, as no CRF data is included in the database.

Safety set

The safety set/population consists of all patients who were randomised and exposed to study medication.

Per-protocol set

All subjects

- without any major protocol violation
- with available values of the relevant study variables

will be included in the per-protocol-set/population.

Comprehensive justification for the classification of a protocol violation as "major" will be given in the integrated clinical study report.

Premature Termination of the study

The sponsor has the right to close this study, and the Principal Investigator has the right to close the centre, at any time, although this can occur only after consultation between involved parties. The IEC/IRB would be informed. If the study/centre had to be closed prematurely, CardioSec GmbH would provide all essential documents necessary for the sponsor's TMF as defined in the GCP Note for Guidance.

The trial will be terminated prematurely in the following cases:

- If AE's occur which are so serious that the risk-benefit ratio is not acceptable.
- If the trial conduct (e.g. recruitment rate, drop-out rate, data quality, protocol compliance) does not suggest a proper completion of the trial within a reasonable time frame.

15 Clinical performance of the study

15.1 Definition of study periods

The study will consist of 4 visits:

Screening visit (V01) lasts from the time point of the patients' arrival at the study centre until assessments 1 hour after end of administration of the investigational products.

The study day 0 comprises patient information, obtaining of age-appropriate assent or written informed consent, checking of in- and exclusion criteria, documentation of demographic data,

documentation of medical history, documentation of pre-existing or concomitant diseases or medication, physical examination of the scalp to verify head lice infestation, decision regarding study inclusion, pregnancy urine test, baseline-assessment of skin and eyes irritation, randomisation, administration of IMP by study staff and assessments for tolerability and questioning on satisfaction with the ≥ 1 hour after end of administration.

Evaluation visit (V02) starts 24 hours (day 1) after end of application. On study visit 2 assessments for tolerability and efficacy will be performed.

Second Treatment visit (V03) starts 7 days (day 7) after end of first application. Assessments for tolerability and efficacy and a second administration of the investigational product will be performed. The second treatment will be performed only in patients without severe irritation of the skin or the eyes in the pre-treatment assessment.

The study ends for the individual patient after he/she underwent the **Follow-up visit (V04)** on day 10, unless any Adverse Event requires further observation.

Patients will be instructed to contact the investigator as soon as possible if they have a complaint or problem with the investigational product so that the situation can be assessed.

All procedures to be conducted during the study, including timing and sequence (as necessary) of all procedures, are indicated in the Flow chart (see chapter 3).

15.2 Study Visits and measures

15.2.1 Screening Visit V01 (day 0): study initiation, registration, enrolment, randomisation, first administration

Patients with suspicion of head lice infestation will be recruited for this study. Written informed consent will be obtained from adult patients or the legal representative of minors. Children able to read and write have to give their assent in written. The assent of younger children will be given verbally as far as appropriate.

Patient's eligibility for participation in the trial will be established by the investigator prior to enrolment.

Demographic data (gender, subject age, hair length (short, shoulder long, mid-back, long), medical history, PTSS during the last week prior to the intended administration of the investigational products, concomitant diseases and concomitant medication (especially chronic medications and use of medications including OTCs during the last 1 weeks, and use of anti-head lice treatments) will be recorded.

Body weight and height will be determined.

The diagnosis head lice infestation will be confirmed by:

- identification of at least five living lice or nymph (immature forms) and five live eggs

The study staff will examine the entire scalp and head hair as described in 15.5.6. Every part of the hair will be examined and the findings will be documented in the CRF:

The severity of head lice infestation will be judged on a 4-point severity scale:

- 0 = no relevant infestation (0-4 lice and/or nymph present);
- 1 = mild (5-9 lice and/or nymphs present);
- 2 = moderate (10-24 lice and/or nymphs present);

- 3 = severe (≥25 lice and/or nymphs present).

Patients will be considered eligible for participation in the study if they do meet any of the inclusion and none of the exclusion criteria. Patients will be received a subject number in ascending order.

In order to ensure administration and precise efficacy and tolerability assessments the patients will be housed at the study site until the assessments 60 minutes after the end of administration have been finished.

The investigational products will be applied at the centre by un-blinded either physicians or specially-instructed nursing staff (see 15.4.

Date and times of start and end of application of the investigational product and times of tolerability and efficacy assessments will be documented.

To sum up, at visit 1 the following operations will be performed and/or parameters controlled by the study team:

- Age-appropriate patient information and obtaining of written informed consent or oral assent
- Documentation of demographic data
- Documentation of medical history
- Documentation of pre-existing or concomitant diseases, PTSS
- Documentation of pre-existing or concomitant medication
- Verifying of head lice infestation, incl. severity
- Checking of inclusion and exclusion criteria including pregnancy test if applicable
- Decision regarding study inclusion, randomisation
- Baseline assessments
- First application of the investigational product
- Post-treatment assessments directly after start of treatment, as well as 1 hour after removing the investigational product
- Questionnaire on the satisfaction with esthetical effect of the products

After the assessments the patient is released from the clinical ward and advised to follow the restrictions made in the protocol and to come back on the next day for the second visit.

15.2.2 Evaluation Visit V02 (day 1): 24h p.a.

24 hours (± 3h) after end of study drug administration the patients will attend the study site. After the 24-hour interval the patient or his/her guardian will perform the efficacy and tolerability assessments.

These data, any concomitant medication taken during the study period, adverse events and any change in concomitant diseases will be recorded in the CRF.

Therefore, at visit 2 the following operations will be performed:

- Hair scalp assessment for living lice (all stages) by study staff
- Assessments of skin and eyes irritation, local tolerability
- Questioning on general well-being
- Questioning on patient's combing

- Registration and documentation of any adverse event and/or changes regarding concomitant diseases, concomitant medication, withdrawal criteria

In case that the examination of the hair and scalp (diagnostic combing) will show active head lice infestation, the life stage of the lice will be documented. The patients will be instructed to use a lice comb until second treatment on day 7.

15.2.3 Second Treatment Visit V03 (day 7)

On study day 7 (V03; +1 day) the scalp and hair will be examined for live head lice by blinded study staff. The life stage of the lice will be documented. The assessments of skin and eyes irritation within 1h prior to administration will be performed. Patients without severe skin or eyes irritations will receive a second treatment with the assigned test product.

The patients will be asked for any concomitant medication taken during the study period and adverse events that occurred.

At visit 3 the following operations are being performed by the study team:

- Hair scalp assessment for living lice (all stages) by study staff
- Second application of the investigational products by the study staff
- Assessments of skin and eyes irritation, local tolerability
- Questioning on well-being
- Questioning on patient’s combing
- Registration and documentation of any adverse events and/or changes regarding concomitant diseases, concomitant medication, withdrawal criteria
- Questionnaire on the satisfaction with the esthetical effect of the products

15.2.4 Follow-up Visit V04 (day 10)

The final visit will be performed on study day 10 (-1; +2 days).

The final visit examination is performed in order to verify if the patient is cured. A patient will be considered cured, if no living head lice could be detected. If living lice present on day 10, the life stages of the lice will be documented. If any other pediculocidal agents employed during the observation period, the patient will be considered a treatment failure.

The following examinations will be performed within the final visit:

- Hair scalp assessment for living lice (all stages) by study staff
- Assessments of skin and eyes irritation, global tolerability, local tolerability
- Questioning on well-being
- Questioning on patient’s combing
- Registration and documentation of any adverse events and/or changes regarding concomitant diseases, concomitant medication, withdrawal criteria

In case that the examination (diagnostic combing) will show active head lice infestation (living nymph or imago), the patient will be considered treatment failure or re-infestation and will be referred to a specialist for further treatment.

Subjects exhibiting either subjective or objective abnormalities when the trial has been completed will be followed up. Any AE which remains unresolved after completion of the trial requires detailed evaluation and follow-up until the AE has been resolved or a reasonable

explanation for its persistence is found. If the subject refuses to follow the instructions of the study staff, the latter is released from responsibility.

At the final visit each trial subject participating in this study will be advised not to participate in any clinical drug studies for a minimum period of two months following this study.

15.3 Restrictions

- No parallel use of any other anti-head lice therapy
- Female patients are to be informed that they have to apply adequate contraceptive methods (e.g. uterine tube sterilisation, sexual abstinence or vasectomised partner or combination of anti-conceptive medication and condom, spermicidal products, diaphragm, temperature method).

15.3.1 Miscellaneous

A pregnancy test in urine will be performed for all female patients with childbearing potential at visit 1 (Screening Visit).

After signing the informed consent patients will receive their subject number, after performing checks for exclusion/inclusion the subjects will receive their random number.

The activities and measurements will be done in the course of the trial as given in the trial flow chart in chapter 3.

For all assessments with exemption of local tolerability, global tolerability, skin irritation and eyes irritation assessment, a deviation up to ± 20 min from scheduled time will be accepted.

For children who are unable for self-assessment and documentation, their guardians will take over these tasks.

15.4 Administration of investigational products

Firstly, a sufficient quantity of the investigational products will be evenly applied by an un-blinded investigator or by specially instructed staff onto dry hair.

The amount applied depends on the length of the patient's hair. For the test product [redacted] the hair should be covered completely with the product.

For GOLDGEIST® FORTE, the following recommendations were given (see table below). Infants will be treated with a maximum amount of 25 mL. However, no more of the investigational product will be used, as necessary to properly cover the hair and scalp.

Category	Hair length	Volume of GOLDGEIST® FORTE applied
1	short hair	about 25 mL
2	hair reaching the patient's shoulders	about 35 – 40 mL
3	hair from patient's shoulders until mid-back	about 75 mL

The test product [redacted] shampoo will be evenly dispensed (massage) in the hair over its full length, (with special diligence on the base of the hair near the scalp). After 10 minutes without

covering the hair a sufficient amount of water will be added to the hair to work up foam. A deviation up to plus 2 minutes due to organisational reasons, particularly to deal with children's ability to follow strict procedures will be accepted. Afterwards the foam will be washed out without shampoo and the hair will be rinsed thoroughly.

The reference product GOLDGEIST® FORTE will be evenly dispensed (massage) in the hair over its full length, (with special diligence on the base of the hair near the scalp). After at least 30 up to 45 minutes without covering the hair, the product will be washed out with warm water. A deviation up to plus 5 minutes will be accepted.

No shampoo must be used for rinsing both investigational products.

Afterwards the hair will be carefully towel-dried. Completely drying the hair using a hair dryer will be performed by the patients themselves or their guardians.

A description of the administration will be given in a specific instruction sheet.

15.4.1 Subject and treatment compliance

The subject's compliance will be monitored during in-house visits by study staff. Any subject found not following directions and restrictions will be withdrawn from the study. Details of the reason for removal of subjects will be reported.

During and after administration of the particular investigational products the patients will strictly follow the instruction of the investigator or study team.

Each investigational product will be administered of one of the study staff. The assessor (blinded study personnel) will check the head of each patient after administration.

15.5 Assessments

Assessments for skin irritation and eyes irritation directly prior to administration (baseline as well as assessments on day 7) will be performed within 1 hour before administration.

Assessments for local tolerability, global tolerability, skin irritation and eyes irritation after administration will be performed directly after start of the treatment plus 5 minutes as well as after the end of administration (the contact time is over and the investigational product is rinsed) within 15 minutes.

For all other assessments a deviation up to ± 20 min from scheduled time will be accepted.

15.5.1 Global tolerability assessment

Global tolerability will be assessed by blinded study staff and by the patient or his/her guardian at day 10. The study staff will perform the rating in each case prior to the assessment by the patient to avoid bias. Afterwards, the patient will self-assess the global tolerability.

The global tolerability will be rated on a 4-category scale with:

Score 1 = very good

Score 2 = good

Score 3 = moderate

Score 4 = poor

15.5.2 Local tolerability assessment

Subjective symptoms (burning, paraesthesia, pruritus) after administration of the investigational products will be rated by the patients on a 4-category scale at day 0 (0h, 1h p.a.) and day 7 (0h, 1h p.a.) directly after study drug application, day 1 (24h), and day 10 p.a.:

Score 1 = no

Score 2 = mild

Score 3 = moderate

Score 4 = severe

It will be investigated, whether the treatments have any influence on the above mentioned subjective symptoms.

15.5.3 Skin irritation assessment

Skin irritation (secondary infection, erythema, excoriation) will be assessed by blinded and trained study staff within 1 hours before first treatment (baseline), as well as directly after study drug application and after the end of treatment on study day 0, day 1, day 7 (pre-treatment, 0h and 1h p.a.) and on day 10 .

For the assessment of skin irritation the following 4-category scale as mentioned above will be used.

Score 1 = no

Score 2 = mild

Score 3 = moderate

Score 4 = severe

It will be investigated, whether the investigational products may cause symptoms of skin irritation.

15.5.4 Eyes irritation assessment

To investigate a potential irritation of the eyes due to the treatment, the trained and blinded study staff will be assessed the severity of redness on both eyes (left, right) within 1 hours before first treatment (baseline), as well as directly after study drug application and after the end of treatment on study day 0, day 1, day 7 (pre-treatment, 0h and 1h p.a.) and on day 10.

For the assessment of eyes the following 4-category scale as mentioned above will be used.

Score 1 = no

Score 2 = mild

Score 3 = moderate

Score 4 = severe

It will be investigated, whether the investigational products may irritate the eyes.

15.5.5 Assessment of esthetical properties

The esthetical properties of the investigational products will be evaluated to determine the satisfaction by the patients with the products after application using a questionnaire about hair and scalp feeling, greasiness, hair look, shininess and volume. The questioning will be performed on day 0 and day 7 after treatment and drying the hair.

1. Your hair does not feels to dry after treatment.

Score 1 = strongly agree

Score 2 = agree

Score 3 = disagree

Score 4 = strongly disagree

2. Your scalp feels pleasant after treatment.

Score 1 = strongly agree

Score 2 = agree

Score 3 = disagree

Score 4 = strongly disagree

3. The greasiness of your hair after treatment is normal.

Score 1 = strongly agree

Score 2 = agree

Score 3 = disagree

Score 4 = strongly disagree

4. Your hair looks good after the treatment.

Score 1 = strongly agree

Score 2 = agree

Score 3 = disagree

Score 4 = strongly disagree

5. The shininess of your hair after treatment is fine.

Score 1 = strongly agree

Score 2 = agree

Score 3 = disagree

Score 4 = strongly disagree

6. The volume of your hair after treatment is fine.

Score 1 = strongly agree

Score 2 = agree

Score 3 = disagree

Score 4 = strongly disagree

15.5.6 Assessment of the hair and scalp (diagnostic combing)

An assessment of the hair and scalp will be performed to determine the number of live lice in the hair with a special lice comb (dry combing). This assessment will be done only for diagnostic reasons. The patients will be instructed to use a lice comb as recommended in the leaflets of the investigational products between the study days. The lice comb will be provided by Oystershell.

For this assessment the hair will be divided into 3 sections: left, middle, and right side of the head. Each section will be combed with a lice comb and the number of live lice encountered per section is recorded. The hair will be assessed for lice on day 0 to determine if the subject complies with the inclusion criterion: ≥ 5 live lice. This procedure will be repeated on day 1, day 7 (pre-treatment) and day 10.

The life stage of lice found during assessments on day 1, day 7 and day 10 must be recorded as this is necessary for determination of re-infestation (see 15.6.1). In case that the examination on day 1, day 7 or day 10 will show active head lice infestation (living nymph or imago), the lice caught by the comb will be discarded. The patients with active head lice infestation on day 10 will be considered a treatment failure and will be referred to a specialist for further treatment.

15.6 Efficacy measurements

The endpoints for efficacy assessment will be based on the cure rate on day 10 p.a. The cure rate is defined as proportion of patients without any live lice, corrected for the rate of re-infestations.

15.6.1 Re-infestation

A re-infestation [22] is defined as:

a) no adult lice or third stage nymphs present following the first treatment AND

b) no more than two adult lice or third stage nymphs found by combing on day 10.

15.7 Safety measurements

15.7.1 Specific safety measurements

Due to the short treatment duration, the topical administration (associated with a low risk of systemic availability of pyrethrum) and comprehensive data on the safety profile of the active substance, special safety investigations (e.g. laboratory safety tests) will not be performed routinely.

No further special safety investigations will be carried out during the study as sufficient information about the investigational products is already available.

15.7.2 Checks for general well-being

The observation phase for PTSS will start one week prior to the first intended study drug administration, for AE's with signing the informed consent, and will end with the release of the subject from the study examination.

Checks for general well-being will be performed in a non-leading manner. In addition to the checks for general well-being at the first visit (0h, 1h p.a.), checks will also be performed, on study day 1 as well as on study day 7 (0h, 1h p.a.) and at the final visit on study day 10.

A registered physician will be available throughout the entire study and all patients will be observed for signs of clinical toxicity during the entire study.

15.7.3 Pre-treatment signs and symptoms and Adverse Events

15.7.3.1 Definition of pre-treatment signs and symptoms and Adverse Events

A **pre-treatment sign and symptom (PTSS)** is defined as any acute existing disease or symptom, which occurs during one week prior to the first intended study drug administration. If, after administration of the investigational product, a deterioration of the intensity of the pre-treatment sign or symptom is observed, this sign and symptom will be documented as an Adverse Event.

An **Adverse Event (AE)** is any unintended or unfavourable sign (including an abnormal finding), symptom or disease occurring in a subject after signing the informed consent until the last study visit, whether or not the event is believed to be causally related to study medication (IMP) or comparative compound. This definition includes any worsening of conditions that were present at the time of entry into the study (signing of the informed consent) (see International Conference on Harmonization (ICH-E2A). AEs occurring after signing the informed consent but before administration of study medication are defined as non-treatment-emergent events. Those events are evaluated separately because in these cases a causal relationship with the study medication can be excluded.

Adverse Drug Reaction: In the pre-approval clinical experience with a new medicinal product or its new usages, particularly as the therapeutic dose(s) may not be established: all noxious and unintended responses to a medicinal product related to any dose should be considered Adverse Drug Reactions. The phrase “responses to a medicinal product” means that a causal relationship between a medicinal product and an Adverse Event is at least a reasonable possibility, i.e., the relationship cannot be ruled out. Regarding marketed medicinal products: a response to a drug which is noxious and unintended and which occurs at doses normally used in man for prophylaxis, diagnosis, or therapy of disease or for modification of physiological function (see the ICH Guideline for Clinical Safety Data Management: Definitions and Standards for Expedited Reporting).

Unexpected Adverse Drug Reaction: An Adverse Drug Reaction, the nature or severity of which is not consistent with the applicable product information e.g. Investigator's Brochure for an unapproved investigational product or package insert/summary of product characteristics for an unapproved product or package insert/summary of product characteristics for an approved product (see: ICH Guideline for Clinical Safety Data Management: Definitions and Standards for Expedited Reporting).

A **Serious Adverse Event (SAE) / Serious Adverse Drug Reaction (Serious ADR)** is defined as any untoward medical occurrence that at any dose

- results in death, or
- is life-threatening, or
- requires inpatient hospitalisation or prolongation of existing hospitalisation, or
- results in persistent or significant disability/incapacity, or
- results in a congenital anomaly/birth defect.

Important medical reactions that may not be immediately life-threatening or result in death or hospitalisation but may jeopardise the patient or may require intervention to prevent one of the other outcomes listed in the definition above should also be considered serious.

Examples of such events are intensive treatment in an emergency unit or at home for allergic bronchospasm, blood dyscrasia or convulsions that do not result in hospitalisation, or development of drug dependency or drug abuse.

The term “life threatening“ in the definition refers to an event in which the patient was at risk of death at the time of the event; it does not refer to an event, which hypothetically might have caused death if it were more severe.

Hospital admission is usually interpreted as requiring at least one overnight stay. A requirement for out-patient treatment in an emergency room is not, by itself, an SAE although the event requiring treatment may be. Elective surgery or other elective procedures which require hospitalisation are not SAEs if the condition being treated or investigated was pre-existing at the time of entry into the study and did not worsen during the study. However, any untoward outcome of any such procedure should be reported as a non-serious AE or an SAE, as appropriate.

Persistent or significant disability or incapacity means that symptoms which disable the subject, defined as a substantial disruption in a person’s ability to conduct normal life functions, do not resolve when study drug is discontinued.
To ensure that there is no confusion or misunderstanding of the difference between the terms “serious” and “severe”, which are not synonymous, the following should be considered: The term “severe” is often used to describe the intensity (severity) of a specific event (as in mild, moderate, or severe myocardial infarction); the event itself, however, may be of relatively minor medical significance (such as severe headache). This is not the same as “serious”, which is based on subject / event outcome or action criteria usually associated with events that pose a threat to a subject’s life or functioning. Seriousness (not severity) serves as a guide for defining regulatory reporting obligations.
Scientific and medical judgment should be used when deciding whether an event is serious.
However, in situations where there is any uncertainty or ambiguity, the event should be managed and reported as if it were serious.

Suspected Unexpected Adverse Drug Reaction – SUSAR is an unexpected Serious Adverse Event causally at least possibly related to the investigational product. If a SAE will be classified as a SUSAR will be decided by sponsor’s drug safety department.

15.7.3.2 AE-Assessment

Each AE will be assessed by an investigator according to the following categories:

Category	Characteristics	Explanations
**Serious**	Non serious	
	Serious	• Results in death<br>• Life threatening<br>• Persistent or significant disability / incapacity<br>• Hospitalisation/Prolongation of existing hospitalization<br>• Congenital anomaly/birth defect
**Expected**	Not applicable	• There is no causal relationship to investigational product assumed.
	No	• Nature, severity, intensity or outcome of the adverse reaction is not consistent with SPC/Investigator's Brochure.
	Yes	• Nature, severity, intensity or outcome of the adverse reaction is consistent with SPC/Investigator's Brochure.
**Intensity**	Mild	• Patient perceives signs or symptoms, but is easily able to tolerate those, his activity is not reduced.
	Moderate	• Signs and symptoms limit patient's normal activities, but don't stop them.
	Severe	• Patient is not able to pursue his normal activities.
**Causal relationship to investigational product**	No relation	Components of assessments of causal relationship<br>• Exposition: Was the investigational product really applied?<br>• Time course: Was the administration of the investigational product followed by the AE in a reasonable time interval?<br>• Probability: Are there other possible reasons for the AE, e.g. concomitant diseases, concomitant medication, environmental or individual factors?<br>• Was the AE terminated or improved after reduction or the stop of medication?<br>• Did the AE reoccur or worsen after reapplication of study drug?
	Possible relationship	
	Probable relationship	
	Certain relationship	
**Action taken regarding study drug**	None	• No change of the planned further application.

Category	Characteristics	Explanations
	Interrupted	• Application was interrupted for a short time.
	Disrupted	• Application was terminated.
	Increased	• Dose was increased.
	Reduced	• Dose was reduced.
	Unknown	• There are no details known.
	Not applicable	• Change of dose was not possible, because for instance application was terminated.
**Outcome**	Recovered/resolved	• The AE is completely subsided. There are no effects felt. Previous status is restored.
	Recovered with sequelae	• The AE is subsided, but patient suffers from symptoms, for instance persistent cough.
	Recovering	• The AE is improved, but patient still feels effects. Recovery is foreseeable.
	Ongoing	• The AE is not resolved, symptoms persist unchanged.
	Fatal	• Patient dies due to the AE.
	Follow-up impossible	• Outcome is unknown because patient didn't come to the follow-up visit. Efforts to get follow-up information failed.

For PTSS only the assessment of intensity will be performed in the same way as for AE.

15.7.3.3 Documentation and Reporting

Documentation of AE or PTSS

Any PTSS or AE, reported spontaneously by a volunteer or observed by the study personnel has to be documented in the CRF regardless if causally related to the investigational product or not.

The event will be described in precise way using common medical terminology. Abbreviations have to be avoided. If possible a diagnosis should made otherwise symptoms will be documented.

The following characteristics of a PTSS have to be documented:

- Description of PTSS
- time of onset and stop time
- severity
- action taken regarding subject
- change to an AE by deterioration

The following characteristics of an AE have to be documented:

- Description of AE
- Time of onset and stop time
- Seriousness
- Expectedness
- Relationship to study drug
- Action taken regarding study drug
- Action taken regarding subject
- Outcome

Reporting of SAE (Serious Adverse Event)

All SAE causally related to study drug or not have to be documented using an additional SAE reporting form. The investigator has to fax SAE report forms within 24 hours after receiving information about the occurrence of an SAE or changed information about a previously reported SAE to sponsor's drug safety.

Drug safety<br>Isabelle Geeraert<br>Fax: + 32-(9) 377 968 0

If the SAE results in death the investigator is obliged to provide further information on request of the ethics committee, the authorities or the sponsor.
An event only classified as a SAE due to hospitalization starts at that time, when the patient arrives at hospital.

The initial SAE report should contain all available information at least:

- Study code
- Patient number
- Date of first dose
- Date of last dose
- Start time
- Description of SAE (diagnosis, symptoms)
- Individual patient data like age, gender
- Concomitant therapy
- Assessment of causality
- Name of investigator

Any effort has to be made to get further information. If new information is received a Follow-up SAE reporting form has to be sent within 24 hours to the drug safety. The case report may be seen as complete if sufficient information regarding causal relationship and outcome is available and no further information is to be expected.

Follow-up of subjects after Adverse Events

The study staff of the clinical facility has to monitor the trial subject's safety from the occurrence of an AE until recovery, return to baseline or a stable state will be achieved. In single cases

follow up of an AE/SAE after last visit can be omitted if there is no causal relationship to study drug and patient's state is improved or stabilised.

Sponsor's responsibility

The sponsor should expedite the reporting to all concerned investigator(s) / institution(s) and to the regulatory authority(ies) of all AE's that are both serious and related. Such expedited reports should comply with the applicable regulatory requirement(s) and with the ICH Guideline for Clinical Safety Data Management: Definitions and Standards for Expedited Reporting.

15.8 Prior and concomitant therapy

Topical use of medication on scalp and hair, prior to the first intended administration of the investigational products, which is listed in the exclusion criteria (see section 14.2), will prevent inclusion into the study.

Treatment of a previous anti-lice treatment with exception of combing the hair using a lice comb within 30 days will prevent inclusion into the study. Treatment with an anti-lice treatment during the study will lead to exclusion of the patient.

Any pre-existing chronic medication taken for treatment of existing disorders and not considered to interfere with the study course or to influence scalp healing will be allowed.

Intake of pre-existing chronic medication used by the patient should be continued throughout the study at a conventional dose and schedule. Their use is to be recorded in the CRF and medical records.

Checks for concomitant medication will be performed at all study visits.

If a patient needs concomitant medication during the study, the responsible investigator should be asked. Concomitant medication is to be documented by brand, type (generic name, if applicable), amount and duration on the CRF. The Principal Investigator / investigator will then decide together with the responsible project manager of CardioSec Clinical Research GmbH, whether the patient has to be excluded.

15.8.1 Concomitant treatment in the event of relevant AEs

Depending on the severity and type of the AEs, the following treatments are suggested treatments and may be changed at the discretion of the responsible physician.

<u>Allergic reaction</u>

Treatment with best standard treatment option according to the local investigator's site circumstances (e.g. antihistamines, glucocorticoids). The investigator will provide all the necessary emergency equipment at the study centre and specially trained staff to handle emergency events during this study (anaphylactic shock).

15.9 Appropriateness of measurements

All methods used for safety assessments are standard methods for which reliability, accuracy and relevance have been documented (e. g. pregnancy test).

15.9.1 Clinical laboratory parameters

In this study no clinical laboratory parameters will be routinely determined.

15.9.2 Clinical parameters

Body weight and height will be measured with commercial instruments. Lice assessment will be done according to the instructions given in chapter 15.5.6. The study staff will be trained for combing and determine the live stages of lice.

15.10 Handling and documentation of clinical data

The Principal Investigator has to ensure that all data required according to this protocol and not mentioned as exception in the following will be entered promptly in the CRF.

The following variables will be documented in the patient's records ore in raw data sheets prior to transfer in the CRF. A paper CRF will be used in this study.

Category	Variables	Source	Details
Identification	Patient number, random number, screening number	Screening/enrolment log, patient file, random list	
Demographic data	Ethnic origin, gender, age, height, weight, participation in another clinical trial within 4 weeks,	Patient file or raw data sheet	
	Childbearing potential, pregnancy, breastfeeding, contraceptive method, result pregnancy test	Patient file or raw data sheet	Woman only
Description of study condition	Confirmation of head lice infestation, severity of head lice infestation, hair length, number of living head lice, live stage of lice	Patient file or raw data sheet	
Randomisation	Check inclusion/exclusion criteria	Patient file or raw data sheet	
	Randomisation	Enrolment log, Patient file	
	Reasons for non-inclusion into treatment	Patient file	
Concomitant medication	Trade name, indication, route, frequency, total daily dose, start date, stop date, ongoing	Patient file or raw data sheet	
Medical history	Disease, date of diagnosis, stop date, ongoing	Patient file or raw data sheet	Relevant findings
Safety	Local tolerability	Patient file or raw data sheet	
	Global tolerability by patient	Raw data sheet	

Category	Variables	Source	Details
	Global tolerability by investigator	Patient file or raw data sheet	prior to patient's assessment
	Skin irritation	Patient file or raw data sheet	
	Eyes irritation	Raw data sheet	
	Satisfaction with the esthetical effect	Raw data sheet	
	Combing by patient	Patient file or raw data sheet	
Administration	Amount of study medication, date of administration, start time, stop time	Drug dispensing log	
Completion	Date of completion, type of termination, reason for premature termination, date of premature termination, health state	Patient file	
AE	Description, start date/time, stop date/time, seriousness, severity, causal relationship, expectedness, action taken regarding study drug, action taken regarding subject	Patient file and raw data sheet	
PTSS	Description, start date/time, stop date/time, severity, action taken regarding subject, deterioration to AE	Patient file and raw data sheet	

All data primarily documented on patient file, raw data sheet, or several logs will be transferred later to the CRF.

For the purpose of quality control, a member of the study team will check data on the CRF’s for formal correctness, completeness and legibility of the entries.

Entries on the CRF’s must be made with a ball-point pen and must be legible. Pencils and correction fluids are not to be used. If corrections are necessary, they will be entered by a member of the study team in the following manner: the wrong CRF entry will be crossed out; however, it must remain legible, and the correct entry will be placed in the correction field (if applicable) or next to the wrong entry. Corrections will be initialled and dated.

A sample CRF is provided in the Trial Master File. The CRF will be supplied by the CRO. The originals are to be returned to the Sponsor. One copy will remain at the Investigator’s site.

The CRFs for any subject leaving the study should be completed at the time when study participation is terminated. CRFs should accurately reflect data contained in subject’s records (i.e. source documents).

15.11 Clinical data management

The data management department of CardioSec GmbH will create a database, which reflects the final version of the CRF. Upon receipt of CRF's in data management, the information from the CRF's will be entered in the validated database according to the corresponding SOP's. Edit checks on completeness, correctness, plausibility (such as range checks, cross-checks) will be performed. All identified discrepancies will be queried by the data manager(s) using data discrepancy forms.

The completed data discrepancy forms will become part of the CRF and, therefore, the original data discrepancy form will be filed with the original CRF; copies must be archived at the centre. All changes to the database will be tracked (audit trail).

Details on data management procedures will be fixed in a detailed data management plan.

16 Biostatistics

The biostatistics department of ACOMED statistik will perform the analysis of the study according to its SOPs.

16.1 Populations for analyses

Safety population (SAF):

All enrolled subjects who administered the investigational product, independently of the duration of treatment.

Per-protocol population (PP):

All enrolled subjects, who show no major protocol deviations (to be defined before database lock). All subjects who provide valuable data.

16.2 Description

Descriptive statistics of all endpoint variables will be tabulated (continuously scaled variables: mean, standard deviation, median, min, max, categorically scaled variables: counts and percentages) by visit and treatment.

16.3 Analysis of primary objective

The cure rate p is defined as proportion of patients who will be found to be cured at evaluation follow-up-visit V04 among all patients received any treatment at visit V01.

The aim of the study is to show superiority for the cure rate of investigated treatment p_T versus a predefined limit of 70%. This limit refers to cure rates found for several formulations which are accepted for the intended use.

The following null hypothesis will be tested:

$$H_{0,prim}: p_T = 70\%$$

If $p_T > 70\%$ and the null hypothesis is rejected by a two sided, one sample χ^2-test at 0.05 level, superiority will be concluded.

16.4 Analysis of secondary objectives

The reference product will be tested in the same way.

Regarding difference of cure rates of test and reference product p_T-p_R, following hypotheses will be tested:

Superiority p_T vs p_R:

$H_{0,sup}$: p_T-p_R=0.

If p_T–p_R > 0% and the null hypothesis is rejected by a two sided, two sample χ^2-test at 0.05 level, superiority of test product will be concluded.

According ICH guideline "Points to consider on switching between superiority and non-inferiority" [23] it is allowed to assess non–inferiority in addition to superiority if superiority cannot be shown. A non-inferiority margin has to be predefined. Moreover, no correction in terms of multiplicity is necessary. As non-inferiority margin δ, a 7.5% worse cure rate will be regarded as clinically not relevant.

The following null-hypothesis will be tested on α-level of 0.025[1].

$H_{0,NI}$: p_T-p_R<δ, whereby δ = -7.5%

The lower, one sided 97.5% confidence interval of difference p_T-p_R will be used for the test. If $H_{0,NI}$ will be rejected, non-inferiority will be shown.

The difference of cure rates p_T-p_R as well the cure rates p_T and p_R will be presented with two sided 95% confidence interval.

The confidence interval for the difference in cure rates is calculated by SAS procedure PROC FREQ using statistic option RISKDIFF.

16.5 Determination of sample size

The design of the trial including the number of subjects is adapted from a previous trial performed with Silcap shampoo [24] and based on statistical calculation.

According to [21], a 90% cure rate can be expected for the study medication. In general, a cure rate of 70% can be assumed to a minimal accepted cure rate. The primary objective refers to superiority of cure rate vs. this limit. A sample size of 42 is required for a one group χ^2-test comparing cure rate of 90% with a fixed limit of 70% (two sided test; alpha-level of 0,05; power=90%; software nQuery advisor 7.0; power=80%: sample size=34).

For the reference group, the identical sample size is used. Assuming 10% drop outs and a 5% rate for re-infestation, the sample size has to be multiplied by 1.1 x 1.05 leading to approximately 50 cases (exactly: 49 cases which have been rounded up to 50 cases).

The sample size is also applicable to show secondary objectives 3 and 4, if following cure rates are assumed:

Superiority: 60% vs 90% (90% power), 65% vs. 90% (80% power).

Non inferiority (margin: -7,5%): 70% vs. 90% (90% power).

---

[1] For practical considerations: Note that the half width of one sided 0.025 one sided CI and two sided 0.05 two sided CI are equal.

16.6 Further statistical analyses

All variables will be tabulated and adequately described according to the respective data type (continuously scaled: mean, standard deviation, median, min, max; categorically scaled: counts and percentages). Results of appropriate statistical tests might be presented in addition. Details analysis and will be fixed in a detailed Statistical Analysis Plan (SAP).

16.7 Statistical software

SAS 9.2 or later versions will be used.

16.8 Quality control for statistical procedures

The statistical analysis will be conducted in compliance with the ICH E9 Note for Guidance on Statistical Principles for Clinical Trials (CPMP/ICH/363/96, 1998).

Quality control will be performed according to the SOPs of ACOMED statistik. Quality control includes checks of data, checks of calculations as well as formal checks of tables, listings and figures. All steps of quality control will be documented with signature of the reviewer and date of review.

17 Evaluation of efficacy parameters

The ITT population will be used for analysis of efficacy variables with exception of non-inferiority, where PP population will be investigated.

The primary analysis will be repeated for PP population.

Missing values will not be imputed patients dropping out because of non-effective treatment will be counted as non-responders by study design.

Details of the analysis will be fixed in the Statistical Analysis Plan (SAP) which will be finalised before database lock.

18 Evaluation of safety parameter

For definition of safety set/population see section 16.1.

All analyses will be performed for the safety population. Due to the short treatment duration, the topical administration (associated with a low risk of systemic availability of pyrethrum or mineral oil) and comprehensive data on the safety profile of the active substance, laboratory safety tests will not be performed routinely.

Secondary parameters for tolerability and safety:

Local tolerability assessment (subjective symptoms)	Burning, paraesthesia, pruritus at day 0 (0h, 1h), day 1, day 7 (0h, 1h) and day 10 p.a.<br>Frequencies and percentages (related to number of patients with information) will be given for each symptom.
Global tolerability assessment (patient):	Patients will assess the overall treatment tolerability at day 10 p.a.<br>Frequencies and percentages (related to number of patients with

	information) will be given for each score as well as for the combined score “very good” / “good”.
Global tolerability assessment (study staff):	The investigator will assess the overall treatment tolerability at day 10 p.a.
	Frequencies and percentages (related to number of patients with information) will be given for each score as well as for the combined score “very good” / “good”.
Skin irritation	Secondary infection, erythema, excoriation of the skin at -1h (baseline assessment), day 0 (0h, 1h), day 1, day 7 (pre-treatment, 0h, 1h) and day 10 p.a.
	Frequencies and percentages (related to number of patients with information) will be given for each symptom
Eyes irritation	Irritation/Redness of the eyes at -1h (baseline assessment), day 0 (0h, 1h), day 1, day 7 (pre-treatment, 0h, 1h) and day 10 p.a.
	Frequencies and percentages (related to number of patients with information) will be given for each symptom.
PTSS, Adverse Events:	Pre-treatment signs and symptoms and treatment emergent adverse events will be described by frequencies on WHO preferred term and system organ class level.

19 Evaluation patient reported outcomes

Secondary parameters:

Satisfaction with the esthetical effect:	The patient will assess the satisfaction with the esthetical effects of the anti-lice product after treatment and hair drying on day 0 and day 7 p.a.
	Frequencies and percentages will be given for each question.

20 Quality assurance

Quality assurance includes all activities undertaken during and after a clinical study to verify and control quality. It embraces internal quality control by the staff itself and by independent second persons as well as monitoring and separate auditing activities. All activities will be performed according to written procedures of the CROs and the facilities involved.

At CardioSec all processes of planning, performance and evaluation are embedded in a comprehensive quality assurance system.

Monitoring of the study will be performed in accordance with the requirements of ICH-GCP.

The monitor is responsible for review of the written Informed Consent, for documentation of ethics committee and ministry of health approvals, for routine review of the CRFs throughout the study, for verifying adherence to the protocol, amendments, study specific SOPs and the completeness, consistency and accuracy of the data being entered on them. All demographic and safety parameters will be 100% verified by the monitor. Furthermore the monitor will review

the TMF at the study site, study drug storage at the visits and check the drug accountability at the close-out visit.

During the interim monitoring visit, the monitor will review the study drug administration, treatment compliance, and the performance of assessments and to determine whether all Adverse Events are appropriately reported within the time periods required by GCP, the protocol, the sponsor, and the German requirements.

The independent QAU of CardioSec GmbH will check the study protocol including informed consent form in order to ensure that these documents are in accordance with GCP standards. The integrated clinical study report will be reviewed by the QAU of CardioSec GmbH and released if methods, procedures and observations are described accurately and are in accordance with the protocol, if potential deviations from the protocol are described thoroughly, and if the integrated clinical study report plausibly reflects the results obtained from the clinical performance, the biometrical report and from the TMF.

Review of Independent Ethics Committee or Institutional Review Board or regulatory inspections (with direct access to source data) are permissible.

In the case of monitor visits or trial audits initiated by the sponsor or by regulatory authorities, the Principal Investigator grant the monitor/auditor access to all documents and source data related to the trial, including all subject files, in order to enable the monitor/auditor to verify all data entered onto the CRFs or any other trial documentation against any source data and documents, while maintaining adequate pseudonymity of the subjects and the confidentiality of the data.

21 Ethics

21.1 Independent Ethics Committee (IEC)

The documents required by the ICH-GCP regulation, the German Drug Law and Medical Device Law including study protocol, informed consent form as well as any subsequent amendment will be submitted to the relevant Independent Ethics Committee for positive vote.

21.2 Ethical conduct of the study

The study will be conducted in accordance with the study protocol, the ethical principles of the Declaration of Helsinki, ICH-GCP guidelines (International Conference on Harmonisation of Technical Requirements for registration of Pharmaceutical for Human Use – Good Clinical Practice), the requirements of the German Drug Law including the GCP regulation, and the German Medical Device Law.

21.3 Volunteer information and consent

The patient will receive a patient information document together with a consent form. The patient information document contains all relevant information about the study in accordance with the GCP requirements.

Every patient has to be informed verbally and also in writing by receiving the patient information document. The written information for patients must be approved by Ethics Committee. The consent form must be signed and dated by the patient prior to the start of the study. The informed consent form must be signed and dated by the physician who conducted the informed consent discussion. The patients must be given a copy of the document.

Every patient has the right to refuse further participation in the study at any time and without giving reasons.

According to the stipulations of the German Federal Data Protection Act, confidentiality and pseudonymity of the patients are assured.

Since minors will be enrolled in this trial, special requirements for this patient group are described in chapter 22.

21.4 Insurance

All subjects participating in the study will have insurance coverage by the sponsor, which is in line with applicable laws and/or regulations

A copy of the insurance certificate (Chubb Insurance Company of Europe SE, insurance number 99497558) has to be available at the study site.

22 Minors enrolled in the study

Children from 1-11 years and adolescents from 12 to less than 18 years can be included in this study.

22.1 Informed consent and assent

As the minor is unable to provide legally binding consent, the legal representative/s has/have to give informed consent on his/her behalf prior to enrolling a minor in the trial. In case both parents are the legal representatives it may be sufficient that at least one parent gives the informed consent, while a letter of attorney of the other parent must be available prior to informed consent process and signing.

The parent(s)/legal representative will be given sufficient time and necessary information to consider the benefits and risks of involving the minor in the clinical trial. Information will be given by experienced personnel to parent(s), or the legal representative on the purpose of the trial and its nature, the potential benefits and risks, and the name of investigators(s) who are responsible for conducting the trial with background professional information (such as education, work experience) and direct contact details (telephone, address, e-mail).

There will not be financial inducement to enrol the minor in the trial; no financial incentive will be offered (other than compensation of expenses and time spent).

Parent(s)/legal representative will be informed of the possibility to revoke informed consent even though it was made in writing, in line with Article 4(d) of the Clinical Trials Directive.

Parent(s)/legal representative will be reassured that the child's treatment will not be prejudiced by withdrawal from the trial, in line with Article 4(d) of the Clinical Trials Directive.

Consent will be obtained from the parent(s)/legal representative before assent is sought from the child, in line with Article 4 (a), (b) and (c) of the Clinical Trials Directive.

Parent(s)/legal representative will be made aware of the rights to refuse participation in a clinical trial and are entitled to withdraw informed consent, without giving reasons.

Parent(s)/legal representatives will be reassured that the withdrawal from the trial will not prejudice the child. Legal representatives who gave informed consent for a child to participate in clinical trials will have the opportunity to follow research as it proceeds (unless clinically inappropriate, e.g., during an operation under general anaesthesia), so as to be able to withdraw the child from the research at any time.

Minors will be involved in discussions and decision-making process only after obtaining consent from the parent(s) or the legal representative, as, where appropriate, the central role of parents should be recognised. An assent (age appropriate) in addition to informed consent of the legal representative is required. If the child's assent is not collected, this will be recorded in the consent form signed by the parents/legal representative and investigator, with the reasons.

Separate information sheets and consent and assent forms will be used in order to provide age appropriate information. Objections raised by a minor at any time during a trial will be considered. The child's will is respected any time, provided it is not considered detrimental to his/her health. The child will not be forced to provide reasons. The legal representative's consent should be checked. The child will be informed of the possibility to withdraw from the trial.

22.1.1 Infants and pre-school children

In this age group, it is not possible to obtain assent and the understanding of research is not expected.
Where the child has some capacity of understanding (pre-school children), age-appropriate information will be provided despite the fact that it will not be possible to obtain assent.

22.1.2 Children of school age (from about 6 years old)

In any case assent, preferably in writing will be obtained when the child is able to read and write, and keeping track of such assent will be performed.

22.1.3 Consent and assent in adolescents

Discretion and professional secrecy vis-à-vis parents when dealing with adolescents binding health professionals will be respected. The specific aspects of disclosure to parents of information concerning adolescents will therefore be taken into consideration for clinical trials in this age group, as well as emancipation status, and age to consent to medical care.

In addition to the requirement for the consent of parents or the legal representative, additional informed consent to participate in clinical trial is required for enrolment of minors of this age group.

When consent is sought from an adolescent who is also a parent of a child to be included in a trial, precautions will be taken to ensure that information provided is sufficiently understood.

22.2 Informed consent (and assent for children) of families with different cultural background

Where appropriate, a cultural mediator independent from the sponsor and investigator, experienced in the language, social habits, culture, traditions, religion and particular ethnic problems will assist in the process of obtaining informed consent and assent.

22.3 Facilities and staff involved

It is ensured that appropriate furniture, toys etc. is available for the short period of duration on site. An experienced paediatrician supervises all activities concerning minors during this trial.

23 Initiation of the trial

The trial will only be initiated when written and dated positive vote by the IEC and approval by the national health authority (BfArM) for the documents required by the GCP regulation including study protocol, informed consent form as well as any subsequent amendments are available.

24 Protocol Amendments

Neither the Investigator nor the Sponsor may modify the protocol without prior written agreement with the other party. Changes in any part of the protocol must be documented in the Study Protocol Amendment. All amendments that would increase the risk to the subject or may alter the results of the study, i.e. increase of the number of subjects, a day dose of study drugs, the age range of study subjects, addition of blood sample(s) or another procedure(s), must be re-submitted to the Institutional Ethics Committee and to regulatory authorities and must be approved before their implementation.

If an amendment to the Study Protocol substantially alters the study design or the potential risks to the subjects, a new subject's consent to continued participation will be needed and approved as stated above.

If the changes in Study Protocol involve only logistical or administrative aspects of the trial (e.g. change of monitor, telephone number), written approvals are necessary from Sponsor, but not from the Institutional Ethics Committee and the Regulatory Authorities before their implementation.

25 Reporting

A summarising report according to ICH standard will be presented to the sponsor including a complete and detailed description of the performance of the study, the biometric planning, the methods and materials, all raw data and calculations for each volunteer, the statistical analysis, summarising calculations, a biometric evaluation and a conclusive assessment of the results.

26 Record keeping

Any documents related to the study must be retained for at least 10 years after the termination of the project or at least two years after the last approval in an ICH region or at least two years after the formal discontinuation of the clinical development. The sponsor will inform the investigator about developments which affect the storage period.

27 Publication policy

On agreement with all parties the results of the studies may be published.

28 Reference list

1. IUCLID dataset on White mineral oil
2. [redacted]
3. [redacted]
4. [redacted]
5. [redacted]
6. Preclinical evaluation, Oystershell
7. Ware, G.W. The pesticide book. Fresno CA: Thomson Publications. 2000. Pp. 65-66
8. Prasada Rao, K.S., C.S. Chetty, and D. Desaiah. 1984. In vitro effects of pyrethroids on rat brain and liver ATPase activities. J. Toxicol. Environ. Health 14: 257-265.
9. V Soni, A Anjikar; Use of Pyrethrin/ Pyrethrum and its Effect on Environment and Human: A Review; PharmaTutor; 2014; 2(6); 52-60
10. Kakko, I., T. Toimela, and H. Tähti. Piperonylbutoxide potentiates the synaptosome ATPase inhibiting effect of pyrethrin. Chemosphere 2000. 40: 301-305
11. Schoenig, G.P. Mammalian toxicology of pyrethrum extract. In Pyrethrum flowers: production, chemistry, toxicology, and uses, ed. J.E. Casida and G.B. Quistad. New York NY: Oxford University Press.1995. Pp. 249-257
12. Karel, A.K. and S.C. Saxena. Investigation on the acute toxic effect of pyrethrum on the blood glucose and of glucose administration on the acute pyrethrum toxicity in MerioneshurrianaeJerdon (Rodentia).Arch. Intern.Physiol.Biochim. 1975. 83:1925.
13. Memo from L.J. Hansen to L. DeLuise. U.S. EPA. Office of Pesticides and Toxic Substances. Pyrethrum extract (technical). Evaluation of a two-generation rat reproduction study to support reregistration of pyrethrum extract. 1991
14. Nash, J.F. A toxicological Review of topical expose to white mineral oils. Food Chem Toxicol. 1996. 34(2):213-25
15. Summary Report 2, EMEA/MRL/362/98-Final Feb. 1998
16. http://toxnet.nlm.nih.gov
17. Dodd CS Intervention for treating headlice. Cochrane Database of Systematic Reviews 2001, Issue 2. Art. No.: CD001165. DOI:101002/14651858.CD001165
18. Feldmeier H. Pediculosis capitis – Die wichtigste Parasitose des Kindesalters. Kinder- und Jugendmedizin 2006; 6:249-59.
19. Letter of equality between [redacted] and Silcap shampoo, June 2014
20. International Guidelines for clinical trials with pediculicides
21. OYS003-10: Clinical Study Report, 2010
22. Burgess, I. F. 1,2-Octanediol, a Novel Surfactant, for Treating Head Louse Infestation: Identification of Activity, Formulation, and Randomised, Controlled Trials PLoS One., 2012

23. ICH guideline: Points to consider on switching between superiority and non-inferiority. http://www.ema.europa.eu/docs/en_GB/document_library/Scientific_guideline/2009/09/WC500003658.pdf, assessed at 13th July .7.2014
24. OYS004-0013: Study Protocol, 2013
